# Supplementary material for: A cross-sectional study of fear of surgery in female breast cancer patients: Prevalence, severity, and sources, as well as relevant differences among patients experiencing high, moderate, and low fear of surgery
Source: PLoS One. 2023 Jun 23;18(6):e0287641. doi: 10.1371/journal.pone.0287641 (PMC10289430; doi:10.1371/journal.pone.0287641)
Supplement: S1 File — (PDF) [file pone.0287641.s001.pdf]

‘**A cross-sectional study of fear of surgery in female breast cancer patients**: Prevalence, severity, and sources, as well as relevant differences among patients experiencing high, moderate, and low fear of surgery’

Sophia Engel[1], Henrik Børsting Jacobsen[1,2], Silje Endresen Reme[1,2]

[1] The Mind Body Lab, Department of Psychology, University of Oslo, Oslo, Norway
[2] Department of Pain Management and Research, Oslo University Hospital, Oslo, Norway

S1 File. Validation of the Norwegian version of the Surgical Fear Questionnaire

Validation process of the Norwegian version of the Surgical Fear Questionnaire

Prior to commencement of the PREVENT trial, the larger trial the present cross-sectional study is part of, the lead principal investigator obtained approval to translate and use the ‘Surgical Fear Questionnaire’ (SFQ) by M. Peters and M. Theunissen, the initial developers of the scale. Moreover, there is an ongoing collaboration between our research group, the Mind Body Lab at the University of Oslo, Norway, and that of M. Peters, the Experimental Health Psychology Research Group at the University of Maastricht, The Netherlands.

Below, a stepwise description of the validation process of the Norwegian version of the SFQ.

Forward backward translation & construct validity

The English version of the ‘Surgical Fear Questionnaire’ (SFQ) was translated in a forward backward procedure by native Norwegian clinical psychologists and experts in the field of clinical health psychology (SER, the principal PI of the present article, as well we HBJ, co-author). After several rounds of forward backward translating the instrument, the final version was derived. Arguments were resolved by discussion. The translating experts regard the final version of the Norwegian SFQ as possessing adequate construct validity.

Convergent & discriminant validity

Convergent and discriminant validity of the Norwegian version of the SFQ were tested by correlating participants’ mean total scores on the instrument with their mean total scores on other priorly validated scales. Based on prior studies, we expected that scores on the Norwegian SFQ would converge well with scores on the Hospital Anxiety and Depression Scale and the item about expected postsurgical pain [1–4]. Moreover, we expected that scores on the Life Orientation Test – Revised, which measures optimism, would negatively correlate with scores on the Norwegian SFQ, thereby confirming the instruments’ discriminative ability. The results of the correlational analyses confirmed these expectations (Table 1). We conclude that the convergent and discriminant validity of the Norwegian version of the SFQ is adequate.

Table 1. Pearson correlations to assess the convergent and discriminative validity of the Norwegian version of the SFQ

	HADS Anxiety	HADS Depression	Expected postsurgical pain	LOT-R
**Norwegian SFQ**	0.507**	0.437**	0.468**	-0.361**
**Range found in other validation studies of the SFQ (incl. references)**	0.498-0.880 [1–3]	0.330-0.832 [1–3]	0.330-0.450 [4]	None

Abbreviations: SFQ, Surgical Fear Questionnaire; HADS, Hospital Anxiety and Depression Scale; LOT-R, Life Orientation Test-Revised
Effect sizes ‘r’: .0 - .3 = small, .3 - .5 = medium, ≥ .5 large
** Correlation is significant at the 0.01 level (2-tailed).

Sophia Engel[1], Henrik Børsting Jacobsen[1,2], Silje Endresen Reme[1,2]

[1] The Mind Body Lab, Department of Psychology, University of Oslo, Oslo, Norway
[2] Department of Pain Management and Research, Oslo University Hospital, Oslo, Norway

Scale reliability

Cronbach's alpha and inter-item correlations were calculated to determine and compare the reliability of the Norwegian version of the SFQ. Cronbach's alpha was 0.894, indicating adequate internal consistency (r ≥0.75). Moreover, Cronbach's alpha remained stable when deleting individual items further supporting the scale's reliability (Table 2). The inter-item Pearson correlation coefficients were mostly moderate (r = 0.3-0.5) or strong (r = ≥0.5), with only one correlation considered as weak (0.0-0.3). All inter-item correlations were significant (Table 3). These results taken together we regard the reliability of the Norwegian version of the SFQ as adequate.

Table 2. Cronbach's α of the Norwegian version of the SFQ and comparable international validation studies of the SFQ

**Cronbach's α of the Norwegian SFQ**	0.894	
**Cronbach's α of the Norwegian SFQ if individual items were deleted**	Item 1	0.870
	Item 2	0.888
	Item 3	0.877
	Item 4	0.880
	Item 5	0.873
	Item 6	0.885
	Item 7	0.878
	Item 8	0.873
**Range of Cronbach's $\alpha's$ found by previous validation studies of the SFQ**	0.813 – 0.934 [1–6]	

Table 3. Inter-item correlation matrix of the Norwegian version of the SFQ

Inter-Item Correlation Matrix

	SFQ1	SFQ2	SFQ3	SFQ4	SFQ5	SFQ6	SFQ7	SFQ8
**SFQ1**	1,000							
**SFQ2**	0,766**	1,000						
**SFQ3**	0,614**	0,457**	1,000					
**SFQ4**	0,526**	0,562**	0,607**	1,000				
**SFQ5**	0,539**	0,395**	0,500**	0,523**	1,000			
**SFQ6**	0,497**	0,338**	0,427**	0,365**	0,509**	1,000		
**SFQ7**	0,444**	0,261**	0,471**	0,381**	0,721**	0,616**	1,000	
**SFQ8**	0,526**	0,345**	0,606**	0,481**	0,641**	0,539**	0,715**	1,000

** Correlation is significant at the 0.01 level (2-tailed).

Sophia Engel[1], Henrik Børsting Jacobsen[1,2], Silje Endresen Reme[1,2]

[1] The Mind Body Lab, Department of Psychology, University of Oslo, Oslo, Norway
[2] Department of Pain Management and Research, Oslo University Hospital, Oslo, Norway

Exploratory factor analysis

Suitability of data

To evaluate the suitability of our data for conducting factor analyses, the Kaiser-Meyer-Olkin (KMO) Measure of Sampling Adequacy and Bartlett’s Test of Sphericity were employed. A KMO score between 0.8 and 1, as well as a significant Bartlett’s Test of Sphericity indicate adequate sampling. The results of our analyses (Table 4) fulfill both conditions. We conclude our data to be suitable to conduct explanatory factor analyses.

Table 4. The Kaiser-Meyer-Olkin Measure of Sampling Adequacy and the Result of Bartlett's Test of Sphericity

Kaiser-Meyer-Olkin Measure of Sampling Adequacy.		0,832
Bartlett's Test of Sphericity	Approx. Chi^2	911,081
	df	28
	p-value	.000

Factor extraction

Factor analysis was conducted using the principal component method, which aims at explaining the largest possible proportion of variance in the original variable. Two components with eigenvalues larger than one were retained and this result was also supported by a scree plot of unrotated eigenvalues (Figure 1).

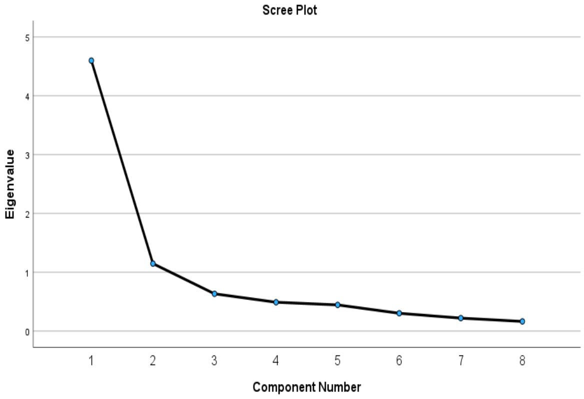

Figure 1. Scree plot of unrotated eigenvalues of the Norwegian SFQ

Orthogonal factor rotation

Varimax rotation with Kaiser Normalization was employed to confirm a simple 2-factor structure of the Norwegian SFQ and to assess the unique contribution of each factor. The rotated two factor solution accounted for 71.8% of the overall variance in the Norwegian SFQ scores (component 1 = 37.9%, component 2 = 33.9%) while preserving the original subscale structure from the English SFQ (Items 1-4 loading strongly on one factor and items 5-8 on another) (Table 5).

The proportion of explained variance of the Norwegian SFQ lies within the range of proportions found by prior validation studies of the SFQ (62.85%-85.2% [1–3,5,6]). It is also within a range that is generally interpreted as acceptable (≥70%).

Table 5. Rotated Component Matrix

	Component	
	1	2
**SFQ7**	0,912	
**SFQ8**	0,802	0,319
**SFQ5**	0,764	0,353
**SFQ6**	0,720	
**SFQ2**		0,904
**SFQ1**	0,345	0,820
**SFQ4**	0,327	0,718
**SFQ3**	0,472	0,632

‘**A cross-sectional study of fear of surgery in female breast cancer patients**: Prevalence, severity, and sources, as well as relevant differences among patients experiencing high, moderate, and low fear of surgery’

Sophia Engel[1], Henrik Børsting Jacobsen[1,2], Silje Endresen Reme[1,2]

[1] The Mind Body Lab, Department of Psychology, University of Oslo, Oslo, Norway
[2] Department of Pain Management and Research, Oslo University Hospital, Oslo, Norway

Conclusion

Based on the analyses described above, we conclude that the Norwegian version of the Surgical Fear Questionnaire possesses adequate construct, convergent and discriminant validity, as well as reliability. Moreover, the results of the exploratory factor analysis confirm, the original two-factor structure of the English SFQ remains preserved in the Norwegian version employed in the present study.

Statistical program

All analyses presented above were conducted using IBM SPSS 29.0.

References

[1] Yang G, Zang X, Ma X, Bai P. Translation, Cross-Cultural Adaptation, and Psychometric Properties of the Chinese Version of the Surgical Fear Questionnaire. Journal of PeriAnesthesia Nursing 2022. https://doi.org/10.1016/j.jopan.2021.08.004.
[2] Bağdigen M, Karaman Özlü Z. Validation of the Turkish Version of the Surgical Fear Questionnaire. Journal of PeriAnesthesia Nursing 2018;33:708–14. https://doi.org/10.1016/j.jopan.2017.05.007.
[3] Garcia ACM, Eduardo AHA, Peters M, Pereira MG, Carvalho EC. Translation, cultural adaptation, and psychometric properties of the surgical fear questionnaire in Brazilian surgery patients 2019.
[4] Theunissen M, Peters ML, Schouten EGW, Fiddelers AAA, Willemsen MGA, Pinto PR, et al. Validation of the Surgical Fear Questionnaire in Adult Patients Waiting for Elective Surgery. PLOS ONE 2014;9:e100225. https://doi.org/10.1371/journal.pone.0100225.
[5] Janković SM, Antonijević GV, Mirković SN, Raspopović KM, Radoičić LR, Putnik SS, et al. Surgical Fear Questionnaire (SFQ) - Serbian cultural adaptation. Vojnosanitetski Pregled 2020;77:1266–70.
[6] Zeleníková R, Kovářová K, Bujok P, Theunissen M. The Czech version of the Surgical Fear Questionnaire: measuring validity and reliability. Central European Journal of Nursing and Midwifery 2022;13:571–8. https://doi.org/10.15452/cejnm.2021.12.0022.
