# Supplementary material for: A cross-sectional study of fear of surgery in female breast cancer patients: Prevalence, severity, and sources, as well as relevant differences among patients experiencing high, moderate, and low fear of surgery
Source: PLoS One. 2023 Jun 23;18(6):e0287641. doi: 10.1371/journal.pone.0287641 (PMC10289430; doi:10.1371/journal.pone.0287641)
Supplement: S2 File — (PDF) [file pone.0287641.s002.pdf]

A cross-sectional study of fear of surgery in female breast cancer patients:

Prevalence, severity, and sources, as well as relevant differences among patients experiencing high, moderate, and low fear of surgery

Sophia Engel, Henrik Børsting Jacobsen, Silje Endresen Reme

23/03/2023

Table of contents

- R setup
- Data loading and data preprocessing
- Multiple imputation
  - Missing value analyses
  - Imputation - setup
  - Imputation - run
  - Imputation - check
  - Conversion of imputed data to long data format
  - Calculation of total scores
  - Converting to MIDS format
  - Dataset with imputation only
- Analyses on imputed data
  - Descriptives
  - T-tests
  - Violin plots
  - ANOVAs
  - Chi-Squares
  - Benjamini-Hochberg correction
  - Venn-diagrams
- Analyses of non-imputed data
  - Descriptives
- Sensitivity analyses
  - Alternative SFQ grouping cut-off
  - Included vs excluded cases

Abbreviations

- SFQ = Surgical Fear Questionnaire

R setup

The following packages have been utilized for the analyses of the present paper:

```
library(tidyverse)          # General Functionalities
library(dplyr)              # General Functionalities
library(haven)              # SPSS dataframe loading
library(EFAtools)           # KMO analysis for SFQ validation
library(lavaan)             # Confirmatory analysis SFQ validation
library(mice)               # Multiple Imputation
library(ggmice)             # Multiple Imputation visualization
library(naniar)             # Missing Completely at Random analysis
library(e1071)              # Skewness and Kurtosis
library(reshape2)           # Merge dataframes
library(gridExtra)          # Merge figures
library(eulerr)             # Venn diagrams
library(lme4)               # Statistics
library(car)                # Statistics
library(rstatix)            # Statistics - games-howell test
library(chisq.posthoc.test) # Statistics - post-hoc chi square
library(tinytex)            # Markdown output
```

Data loading and preprocessing

Data loading

Data was loaded as an SPSS datafile (.sav). The code below only serves for illustrative purposes to protect privacy sensitive information. The dataset contains 74 variables and data of 195 participants.

```
data <- read_sav("Drive:/Path/Filename.sav")
```

Data preprocessing

Variables were classified as continuous (numeric) or categorical (factor).

```
# Variable class assignment
col_fac <- c("MaritalSt2G","Children","Educ2G","OccupationalStatus","PreviousCance
r","Psychiatric","PsychDrugs","PriorBreastSurg","Diagnosis","Neoadjuvant","Mastectom
y","BreastReconstruction","BCTOnco","BCTOnly","SN","ALND","Hospital","NRS1","NRS5","N
RS9","NRS13","NRS17","NRS2","NRS6","NRS10","NRS14","NRS18")

for (i in col_fac){data[[i]] <- as.factor(data[[i]])}

col_num <- c("Age","PainExpectation","PriorSurg","LOT_R1","LOT_R2","LOT_R3","LOT_R
4","LOT_R5","LOT_R6","LOT_R7","LOT_R8","LOT_R9","LOT_R10","SFQ1","SFQ2","SFQ3","SFQ
4","SFQ5","SFQ6","SFQ7","SFQ8","HADS1","HADS2","HADS3","HADS4","HADS5","HADS6","HADS
7","HADS8","HADS9","HADS10","HADS11","HADS12","HADS13","HADS14","IEQ1","IEQ2","IEQ
3","IEQ4","IEQ5","SocSup1","SocSup2","SocSup3","SocSup4","SocSup5","SocSup6")

data[col_num] <- sapply(data[col_num],as.numeric)
```

Multiple imputation

Multiple imputation procedures were based on the book ‘Flexible Imputation of Missing Data’ (second edition) by van Buuren (1) and ‘Applied Missing Data Analysis with SPSS and (R)studio’ by Heymans et al. (2). The imputation incorporates the R packages mice (version 3.14.0) and ggmice (version 0.0.1).

1. Buuren S van. Flexible Imputation of Missing Data, Second Edition. CRC Press; 2018. 444 p.
2. Book_MI.knit [Internet]. [cited 2022 Jun 11]. Available from:https://bookdown.org/mwheymans/bookmi/ (https://bookdown.org/mwheymans/bookmi/)

Missing value analyses

First the total amount, as well as patterns of missing values were analyzed. Here, 5 variables were excluded because of allowed missing values. These all relate to items of the numeric rating scale.

```
Tot_Pers_NA <- (sum(is.na(data[-c(27:31)]))/prod(dim(data[-c(27:31)])))*100
NA_column_sums <- data.frame(colSums(is.na(data))/nrow(data))*100
```

There were no structural patterns in missing values observed.

```
ggmice::plot_pattern(data[-c(27:31)], square=FALSE, rotate=TRUE, cluster=NULL)
```

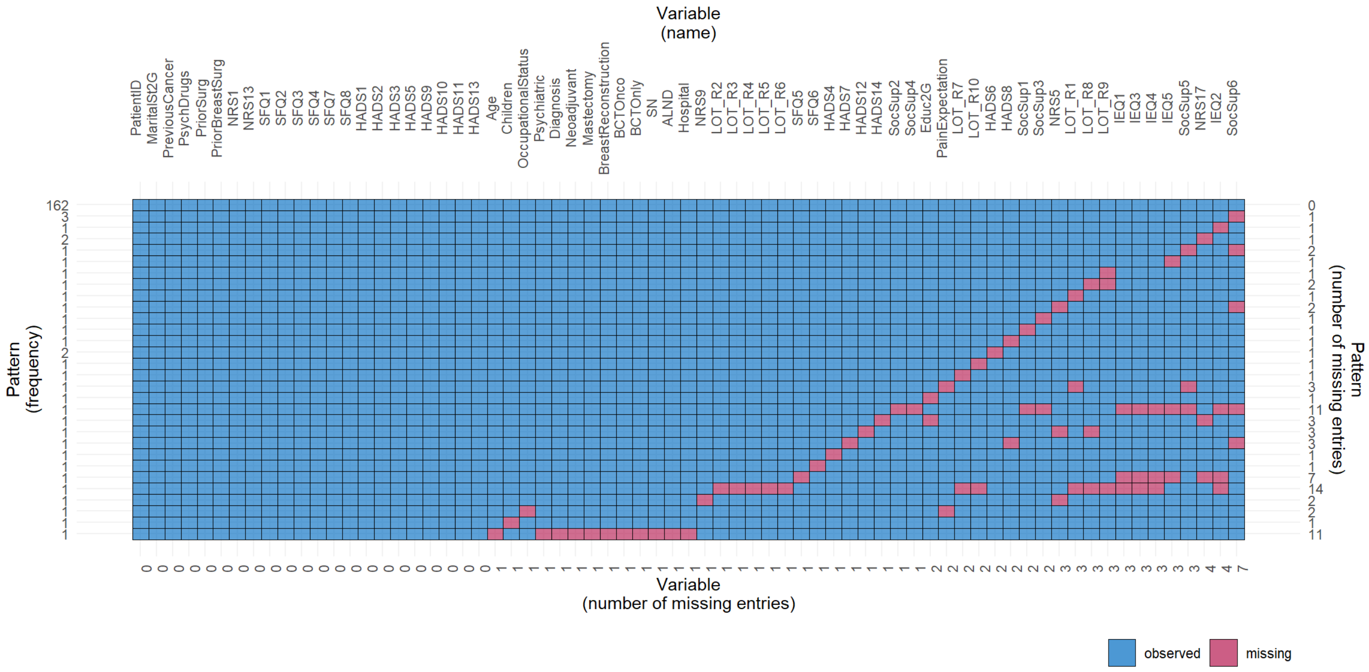

Missing Completely At Random analysis (MCAR) confirmed that data were missing completely at random (insignificant MCAR test).

```
mcar_test(data[-c(1,27:31)])
```

Imputation setup

Next, an empty imputation was run to obtain a prediction matrix. The presence of warning messages was checked using loggedEvents.

```
imp <- mice(data, m=1, maxit=0)
imp$loggedEvents
```

The prediction matrix displays the imputation method by which ‘R’ will replace missing values for each variable. For the present study the prediction matrix was adapted in four ways:

- First, the ‘quickpred()’ function employing a Pearson correlation coefficient set at 0.1 (default) was used to ensure that only variables that correlate with each other would be used to predict each others missing values.

- Second, ‘age’ was set as a fixed predictor that would be used in all predictions.
- Third, variables that were not needed to be imputed (for example ‘subject ID’) or not expected to have missing values (like for example conditional items of questionnaires) or multi-collinear with other items, were excluded from the imputation procedure (by setting them 0 horizontally and vertically in the matrix).
- Finally, the prediction method that ‘R’ assigned to each variable was checked and adapted where necessary. In accordance with common practice, dichotomous variables were imputed by means of logistic regression (‘logreg’), categorical variables with more than two levels using polytomous regression (‘polyreg’) and continuous variables with predictive mean matching (‘pmm’).

```
# Automatic prediction matrix based on correlations
pred_mat <- quickpred(data)

# Always included in imputation
pred_mat[,'Age'] <- 1
pred_mat['Age',] <- 1

# Exclude from imputation (h+v = 0)
excl_MI <- c('PatientID','NRS2','NRS6','NRS10','NRS14','NRS18')
pred_mat[,excl_MI] <- 0
pred_mat[excl_MI,] <- 0

# Check imputation method
imp_method <- data.frame(imp$method)
method_list <- c("","pmm","","logreg","logreg","logreg","","logreg","","","","polyre
g","logreg","logreg","logreg","logreg","logreg","logreg","logreg","logreg","pm
m","","logreg","logreg","","logreg","","","","","","pmm","pmm","pmm","pmm","pmm","pm
m","pmm","pmm","pmm","pmm","","","","","pmm","pmm","","","","","","pmm","","pmm","pm
m","pmm","","","","pmm","","pmm","pmm","pmm","pmm","pmm","pmm","pmm","pmm","pmm","pm
m","pmm","pmm")

method_check = cbind(method_list,NA_column_sums)
```

Imputation run

After these preparations, the full imputation procedure was re-run. This time using 100 imputations with 20 iterations each (based on recommendations (1)). The seed argument (set at ‘1’) was used to ensure repeatability of the imputations.

```
imp_run <- mice(data, m=100, maxit=20, pred = pred_mat, method = method_list,
                printFlag = T, seed = 1)
```

Imputation check

Before commencing statistical analyses, the fit of the imputation model was checked. Convergence plots showed healthy convergence and density plots plausible imputed values.

To visualize convergence, two graphs were constructed per variable. One showing the mean and the other the standard deviation of imputed values. Generally, good convergence is achieved when streams do not display any trends, and the variance between streams is about as large as that within a given stream. As the graphs show, these criteria were fulfilled for all variables for which missing values were imputed in the present study.

Density plots display the kernel densities of observed (blue) and imputed (red) values. Within a good fitting imputation model, density plots of observed and imputed data should have similar shapes. This was the case in the present study.

```
plot(imp_run)
```

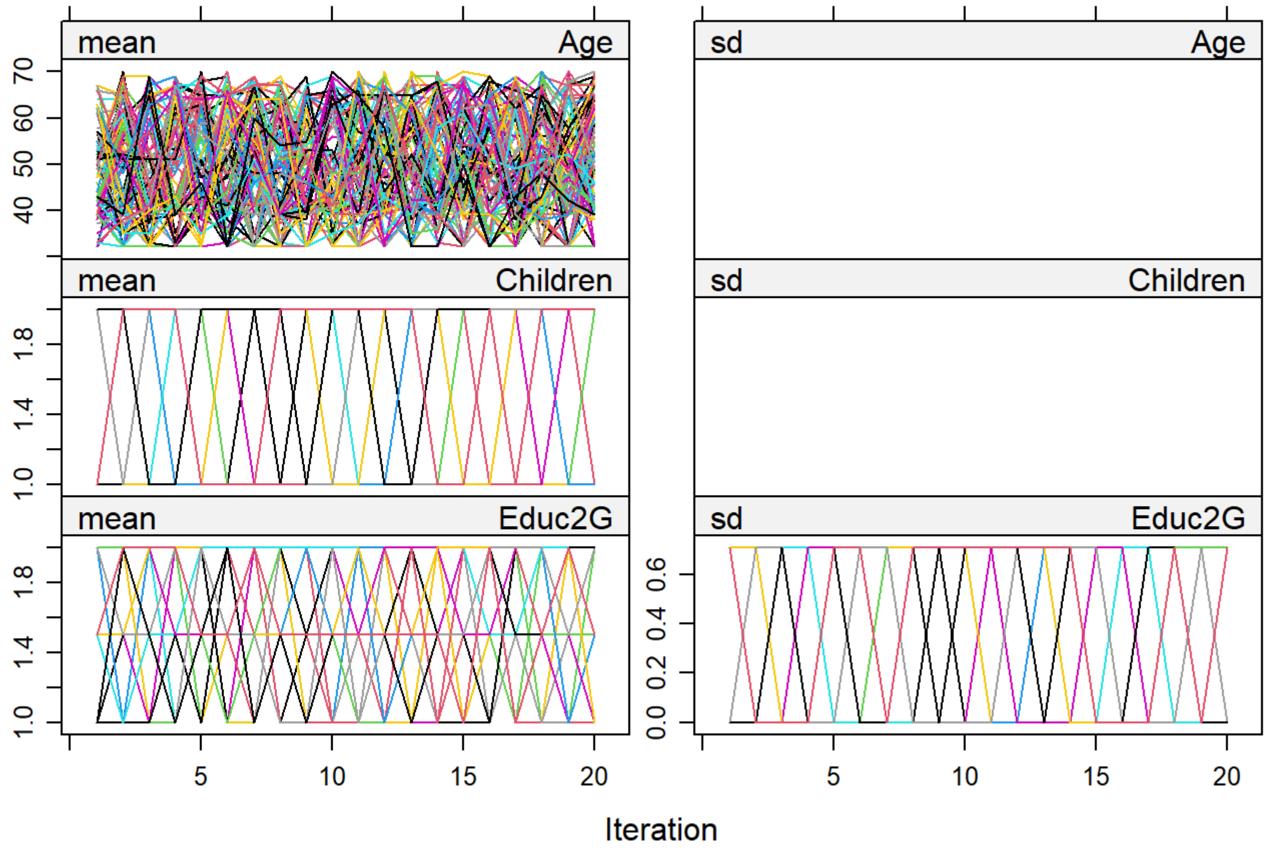
mean
Age
sd
Age
mean
Children
sd
Children
mean
Educ2G
sd
Educ2G
Iteration

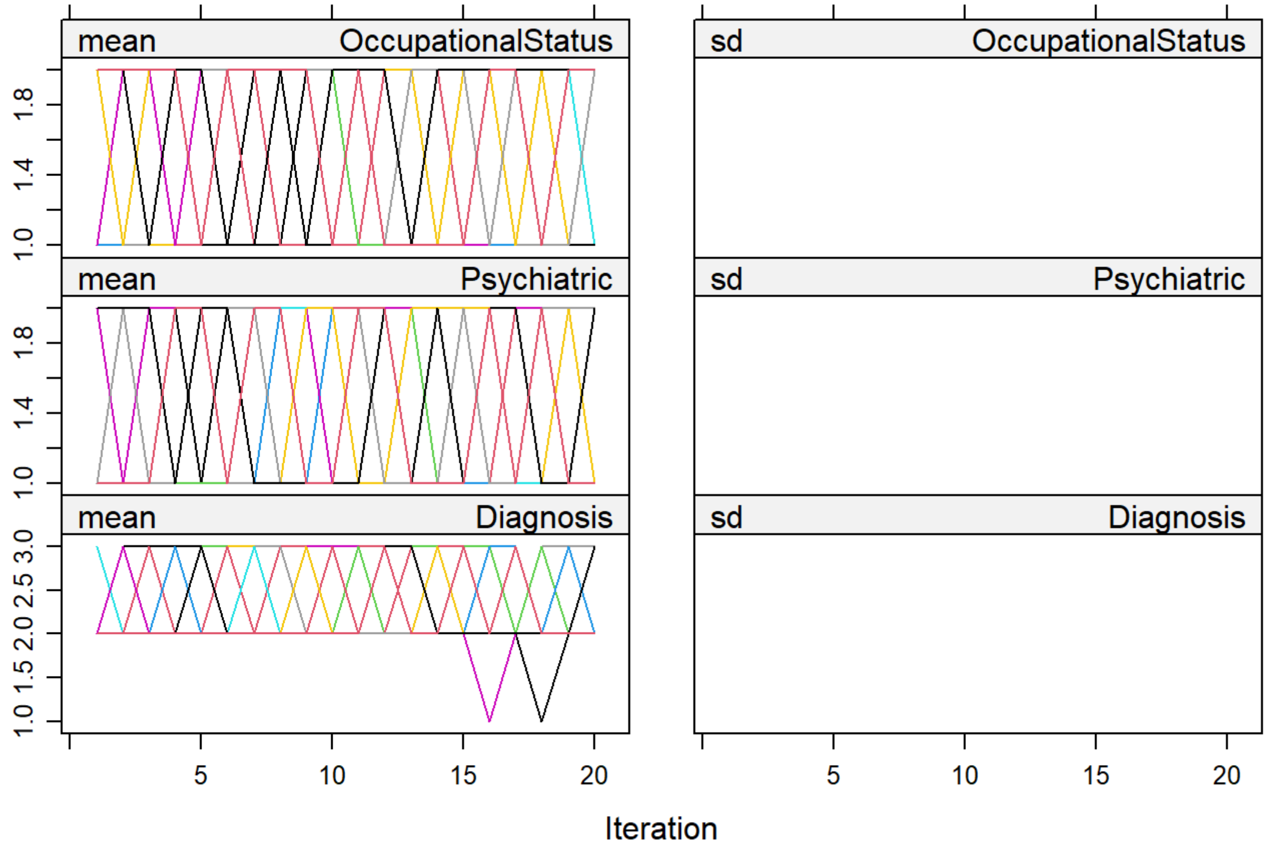
mean
OccupationalStatus
sd
OccupationalStatus
mean
Psychiatric
sd
Psychiatric
mean
Diagnosis
sd
Diagnosis
Iteration

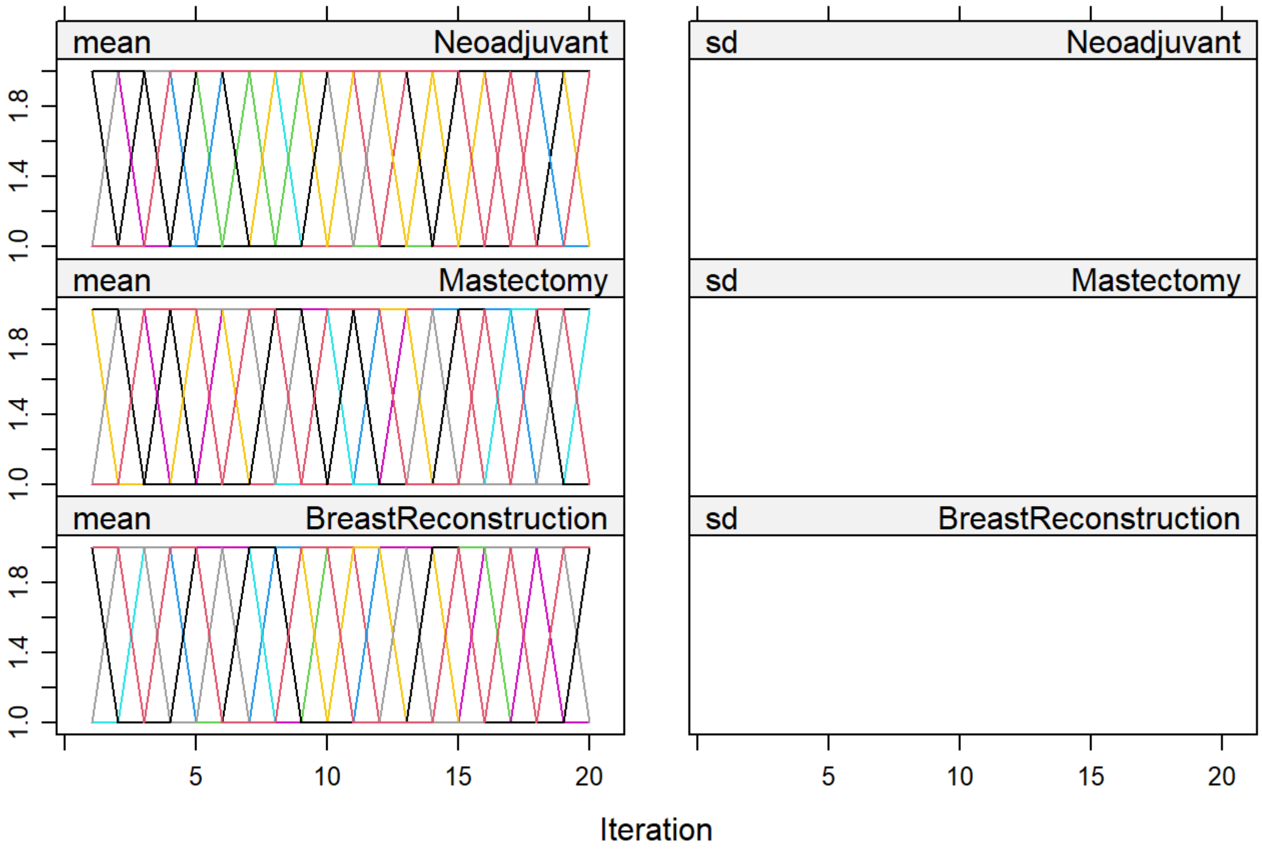
mean
Neoadjuvant
sd
Neoadjuvant
mean
Mastectomy
sd
Mastectomy
mean
BreastReconstruction
sd
BreastReconstruction
1.0
1.4
1.8
5
10
15
20
Iteration

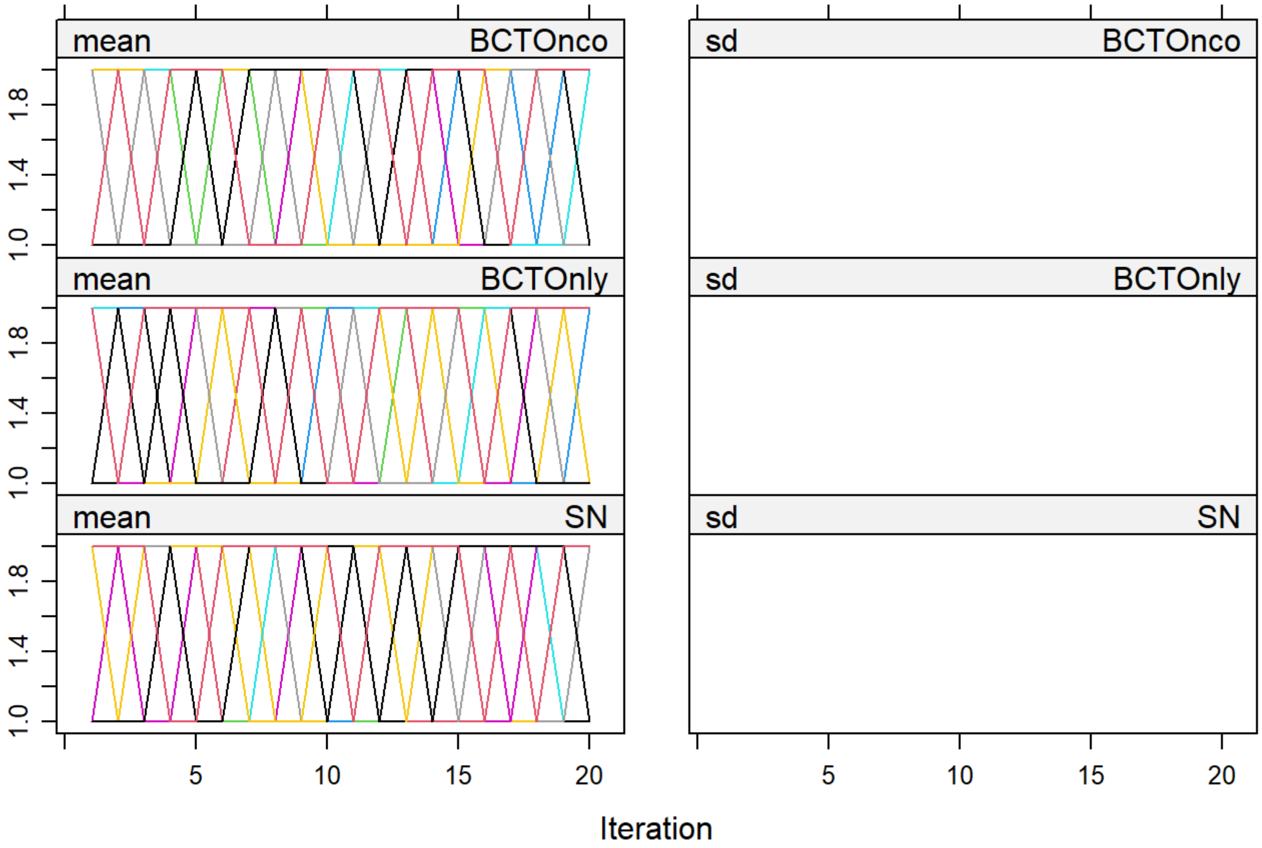
mean
BCTOnco
sd
BCTOnco
mean
BCTOnly
sd
BCTOnly
mean
SN
sd
SN
1.0
1.4
1.8
5
10
15
20
Iteration

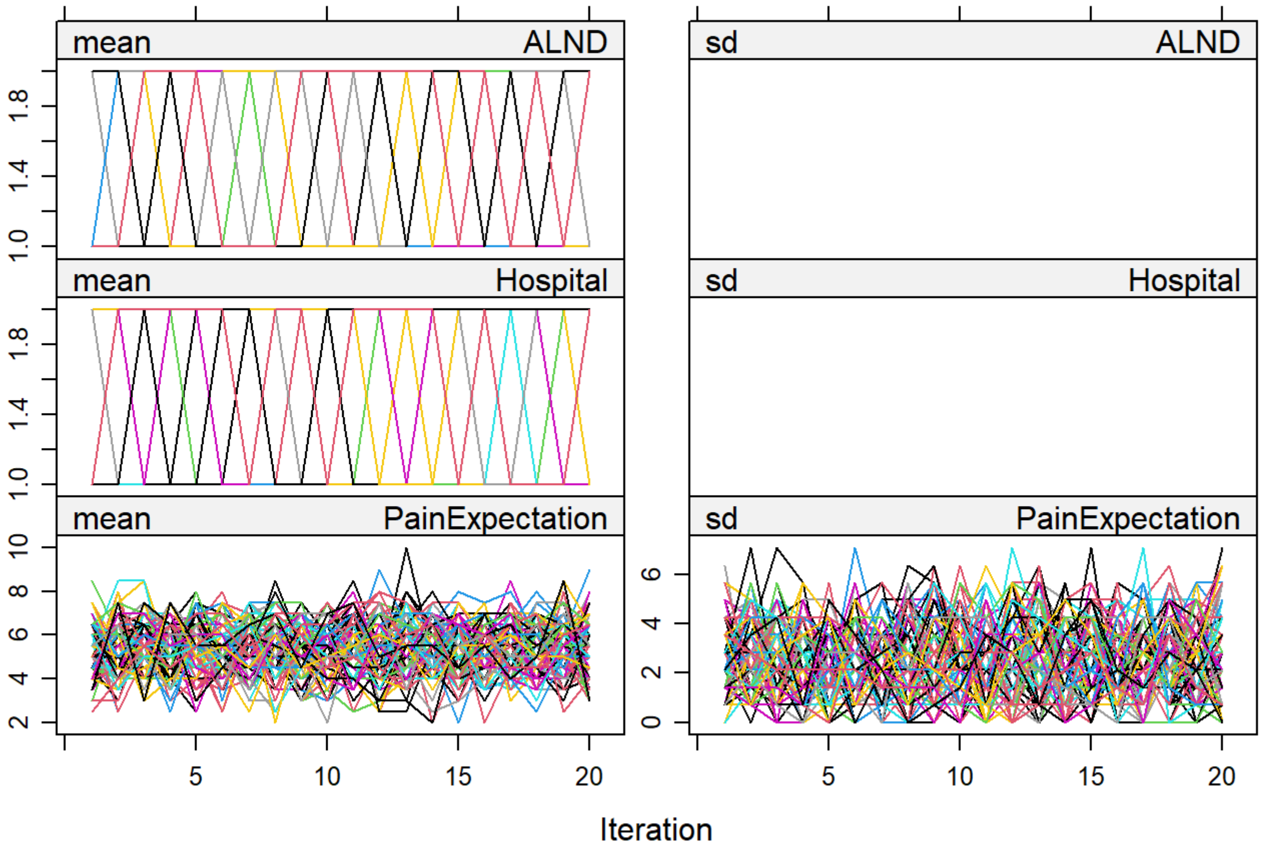

mean
ALND
sd
ALND
mean
Hospital
sd
Hospital
mean
PainExpectation
sd
PainExpectation
Iteration

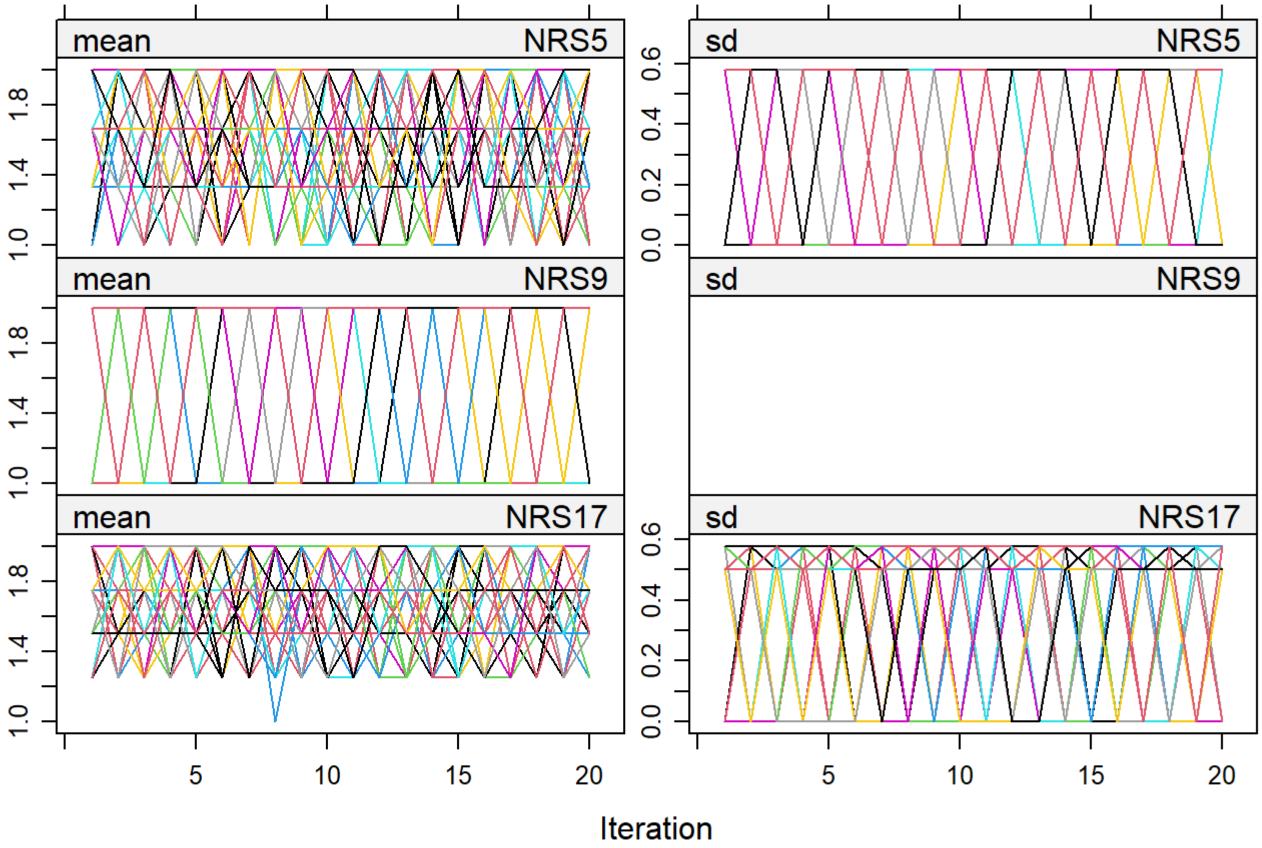

mean
NRS5
sd
NRS5
mean
NRS9
sd
NRS9
mean
NRS17
sd
NRS17
Iteration

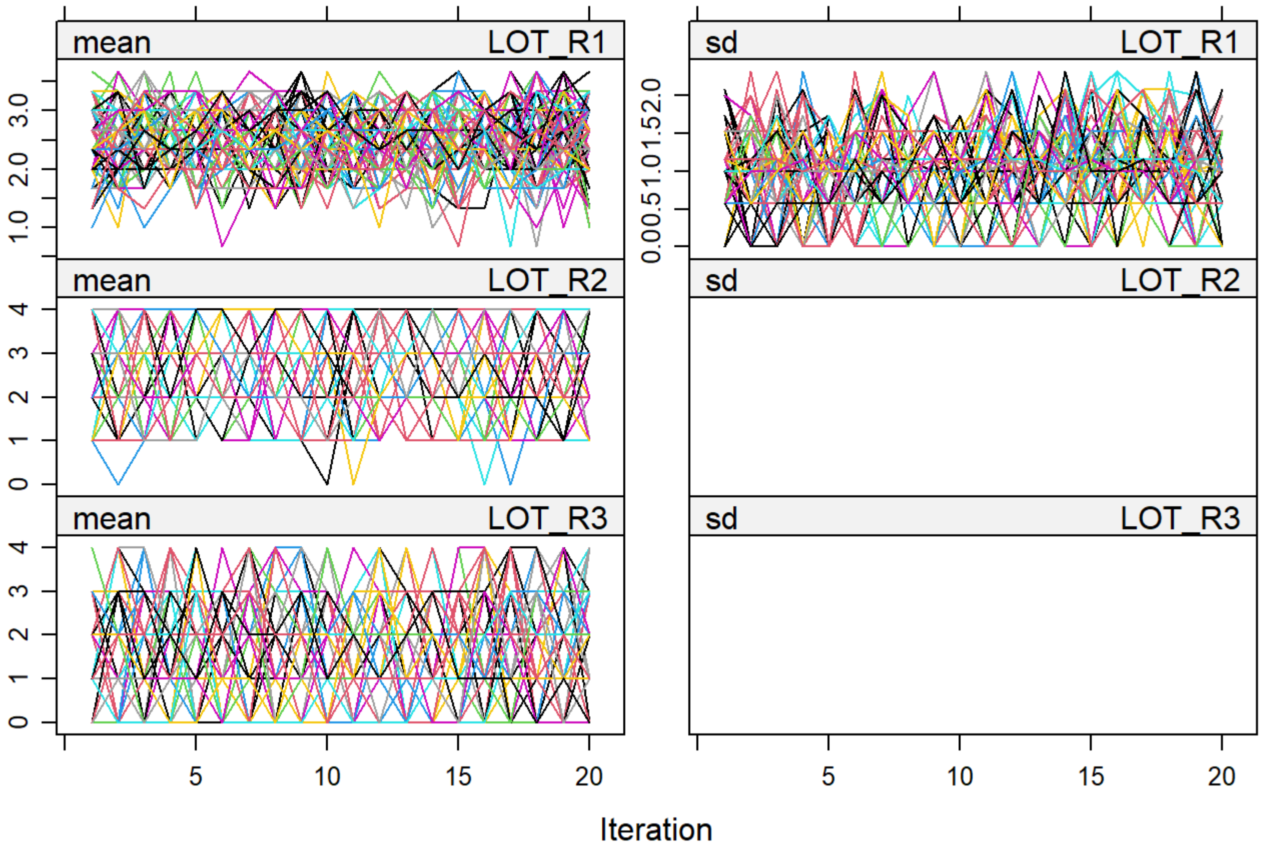
mean
LOT_R1
sd
LOT_R1
mean
LOT_R2
sd
LOT_R2
mean
LOT_R3
sd
LOT_R3
Iteration

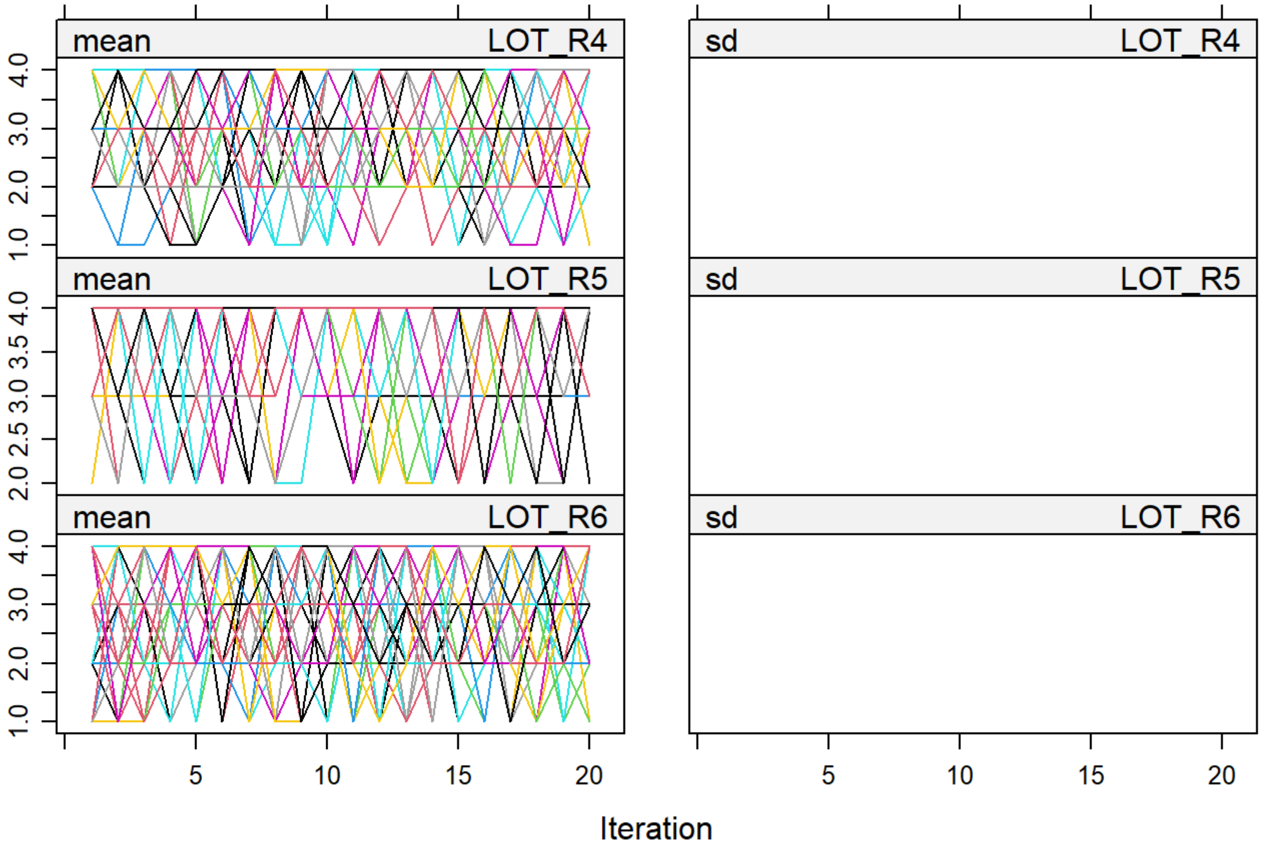
mean
LOT_R4
sd
LOT_R4
mean
LOT_R5
sd
LOT_R5
mean
LOT_R6
sd
LOT_R6
Iteration

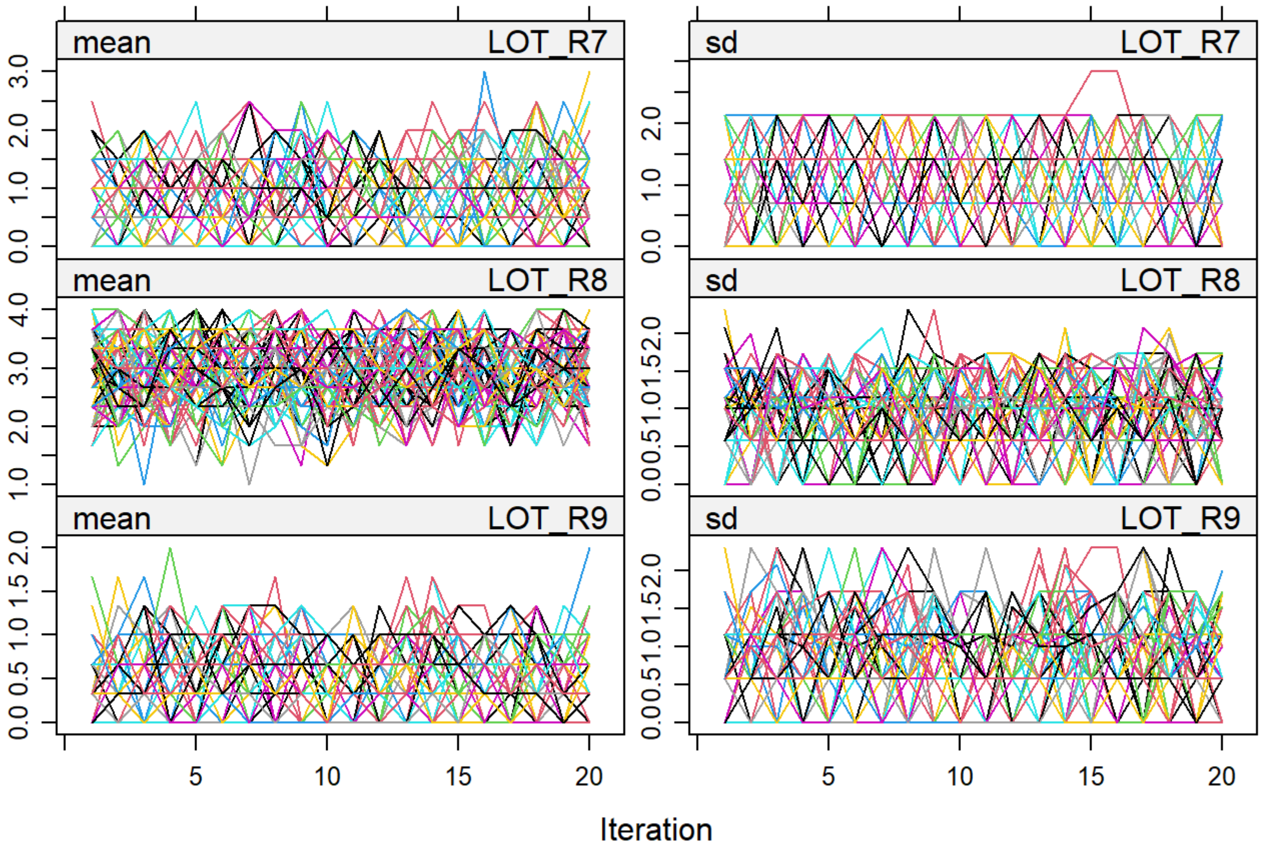
mean LOT_R7
sd LOT_R7
mean LOT_R8
sd LOT_R8
mean LOT_R9
sd LOT_R9
Iteration

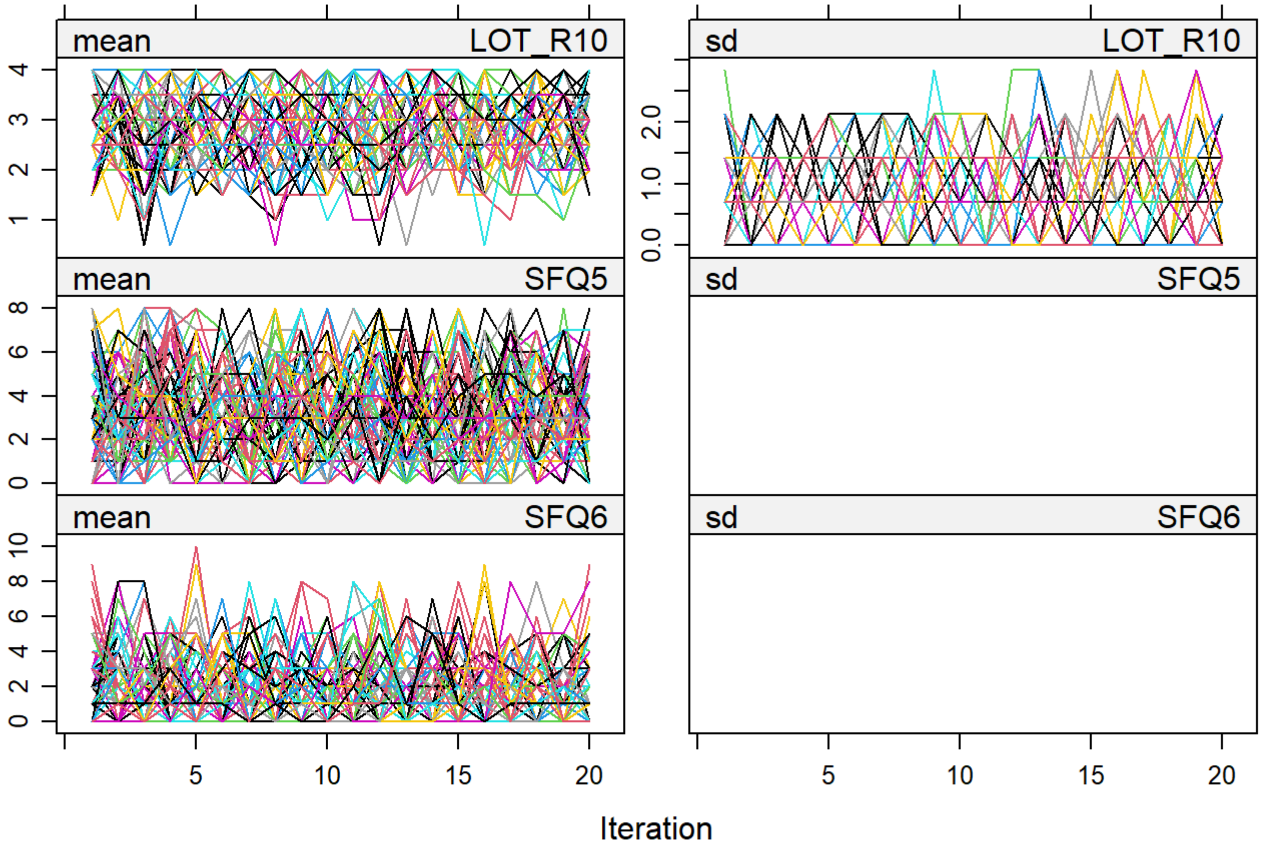
mean LOT_R10
sd LOT_R10
mean SFQ5
sd SFQ5
mean SFQ6
sd SFQ6
Iteration

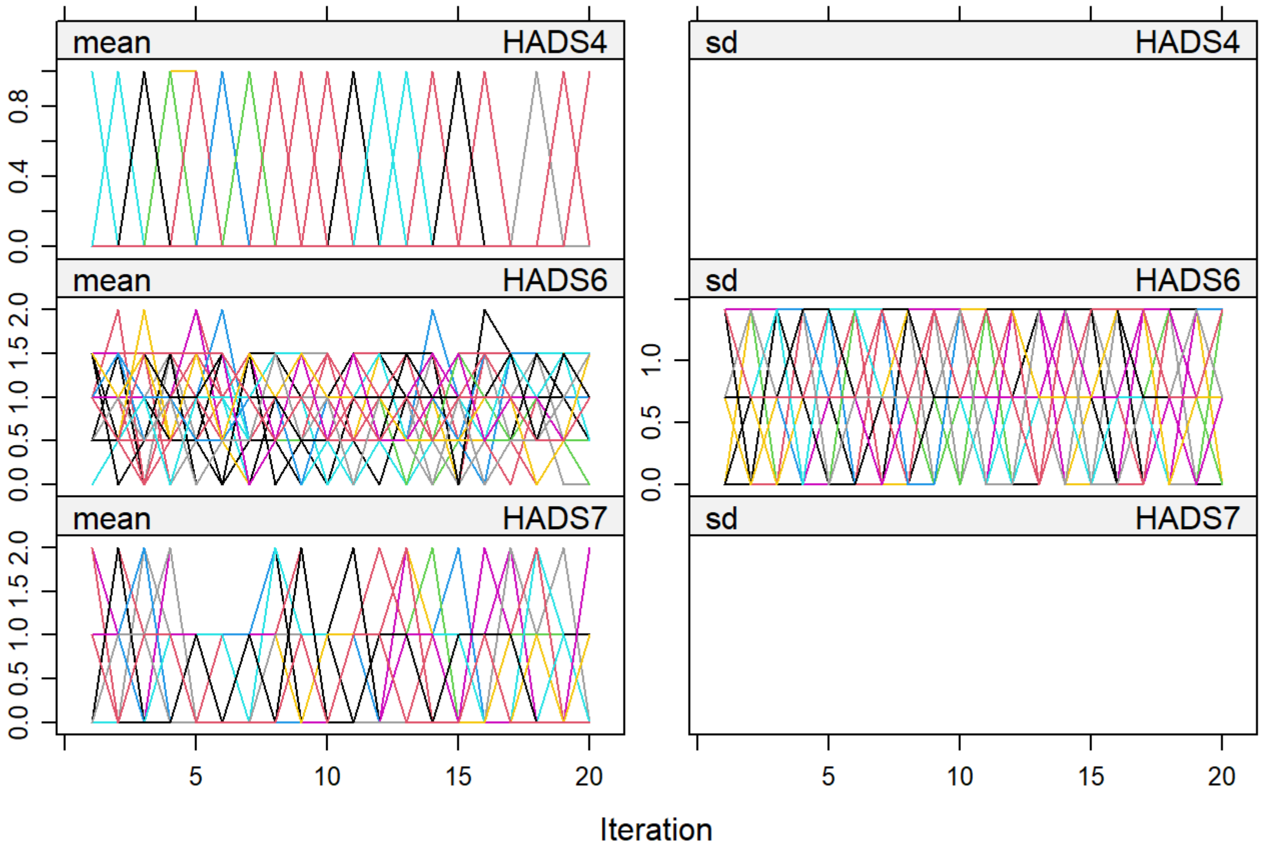
mean
HADS4
sd
HADS4
mean
HADS6
sd
HADS6
mean
HADS7
sd
HADS7
Iteration

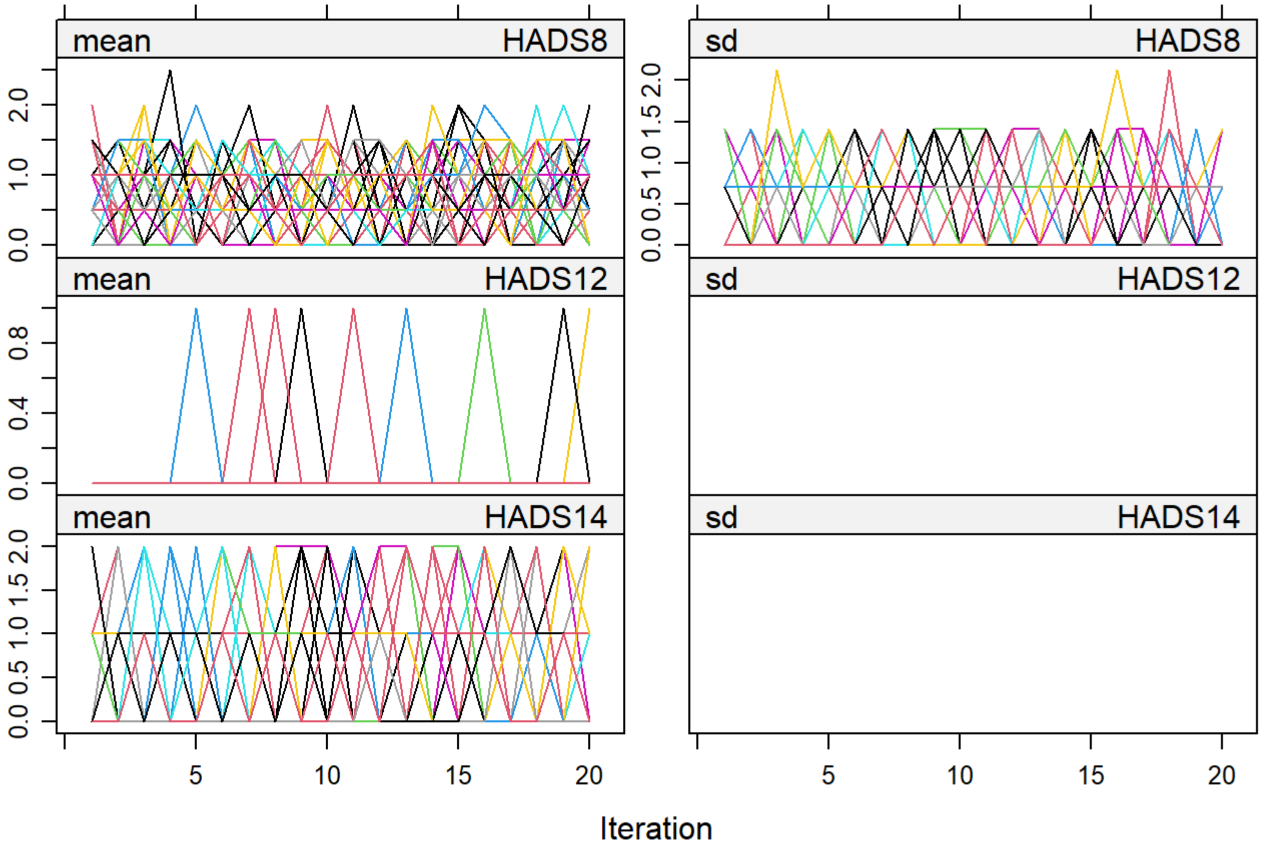
mean
HADS8
sd
HADS8
mean
HADS12
sd
HADS12
mean
HADS14
sd
HADS14
Iteration

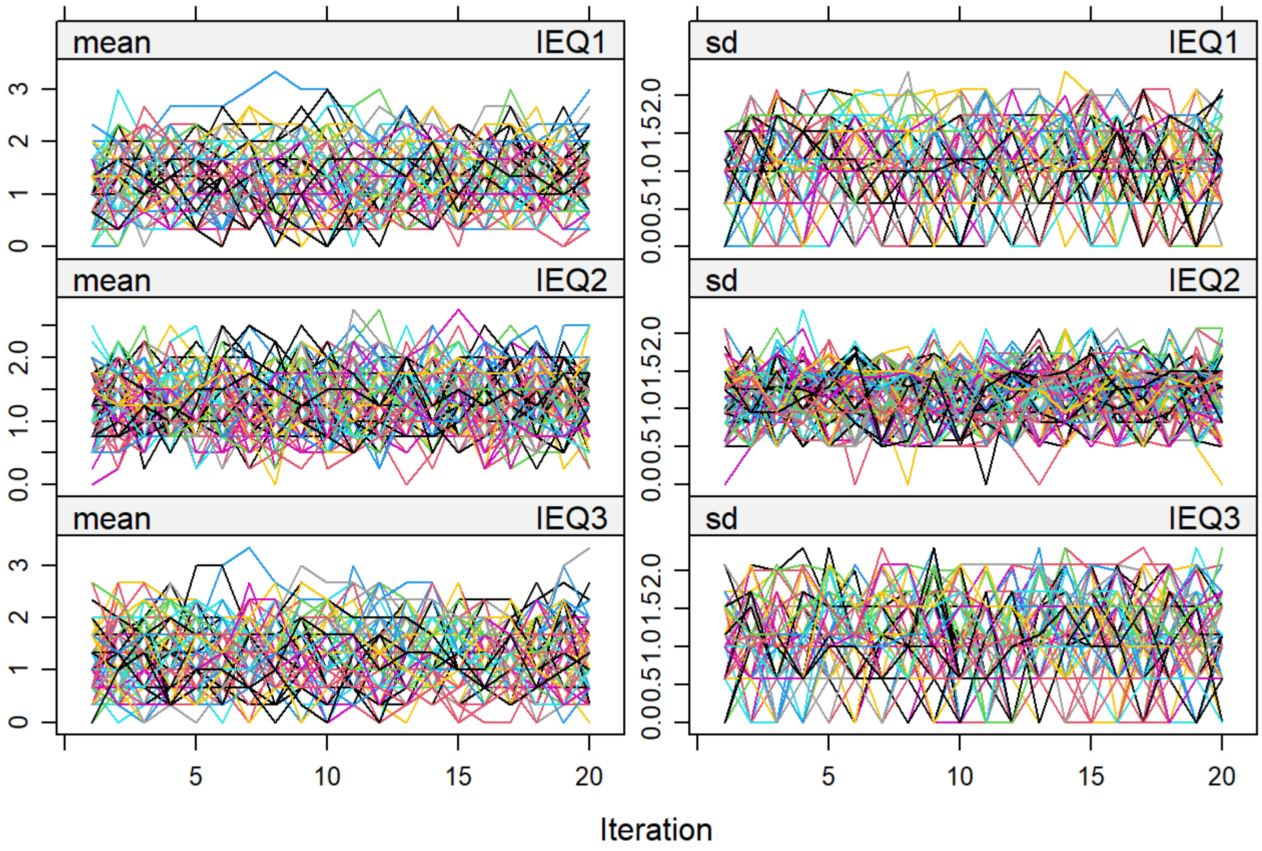
mean
IEQ1
sd
IEQ1
mean
IEQ2
sd
IEQ2
mean
IEQ3
sd
IEQ3
Iteration

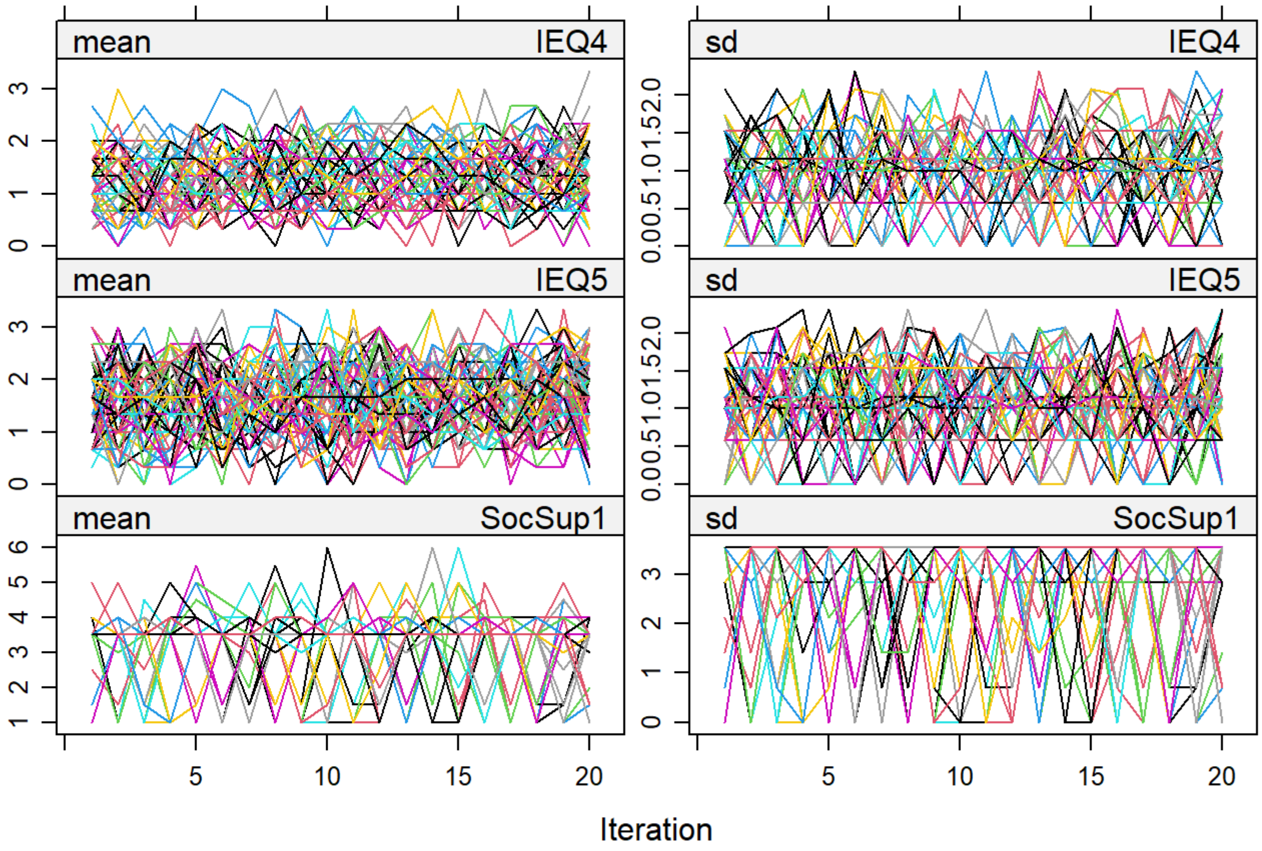
mean
IEQ4
sd
IEQ4
mean
IEQ5
sd
IEQ5
mean
SocSup1
sd
SocSup1
Iteration

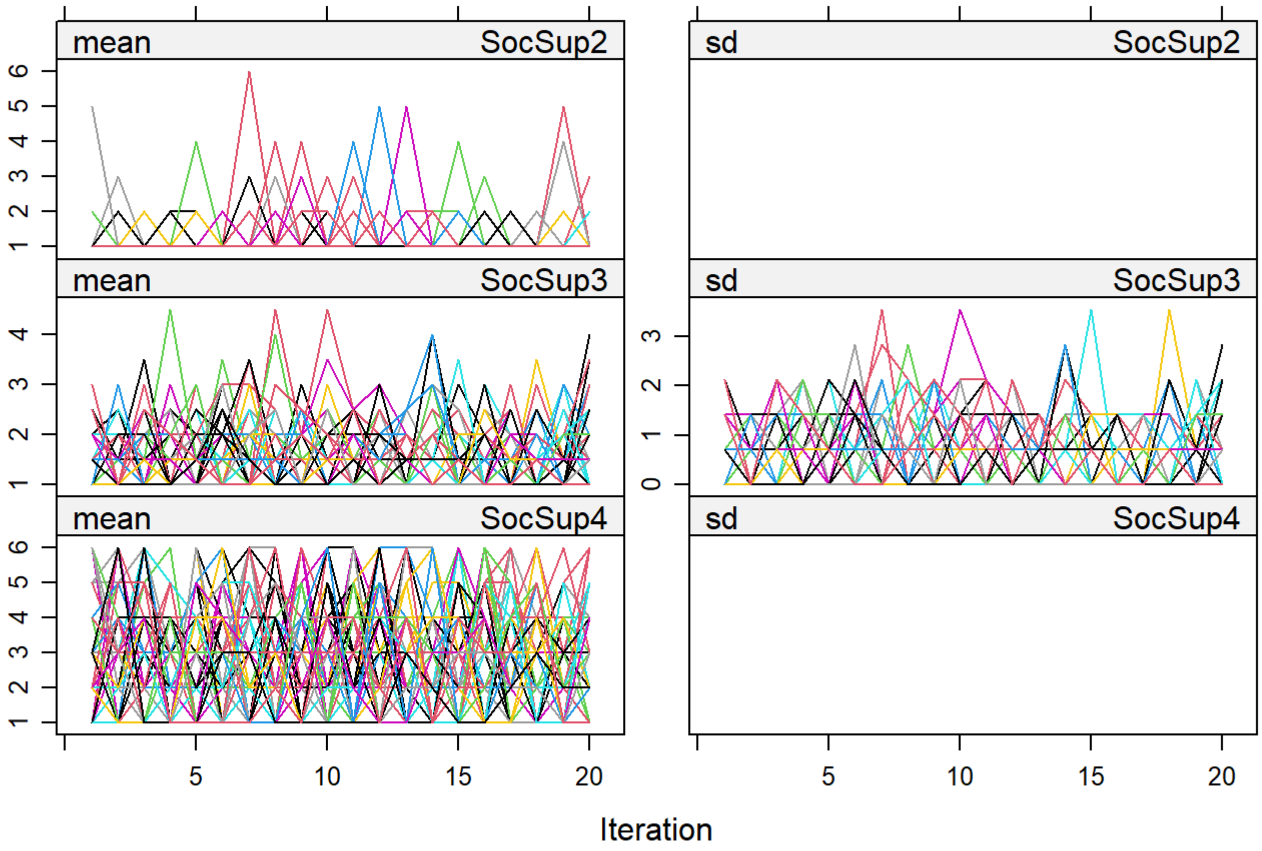

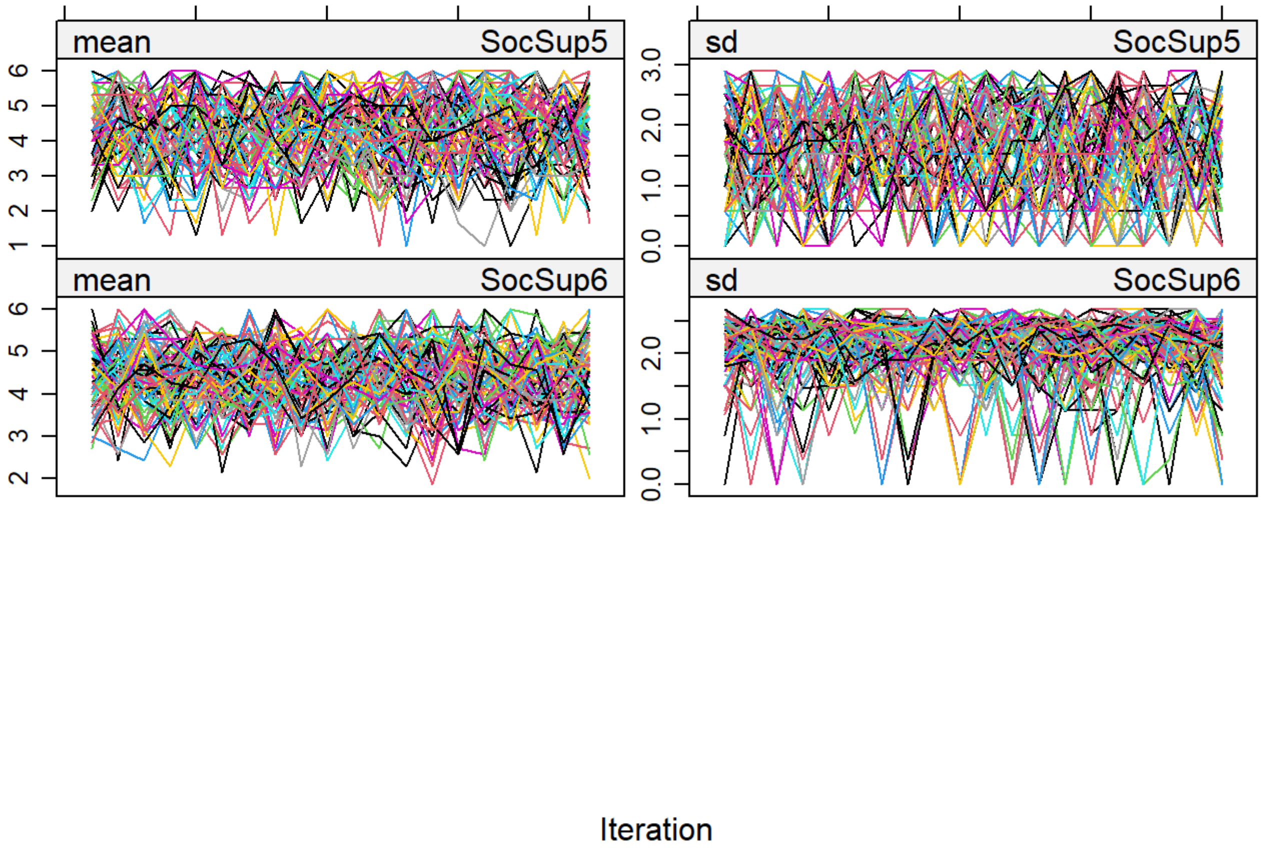


```
densityplot(imp_run, ~ LOT_R1+LOT_R7+LOT_R9+LOT_R10)
```

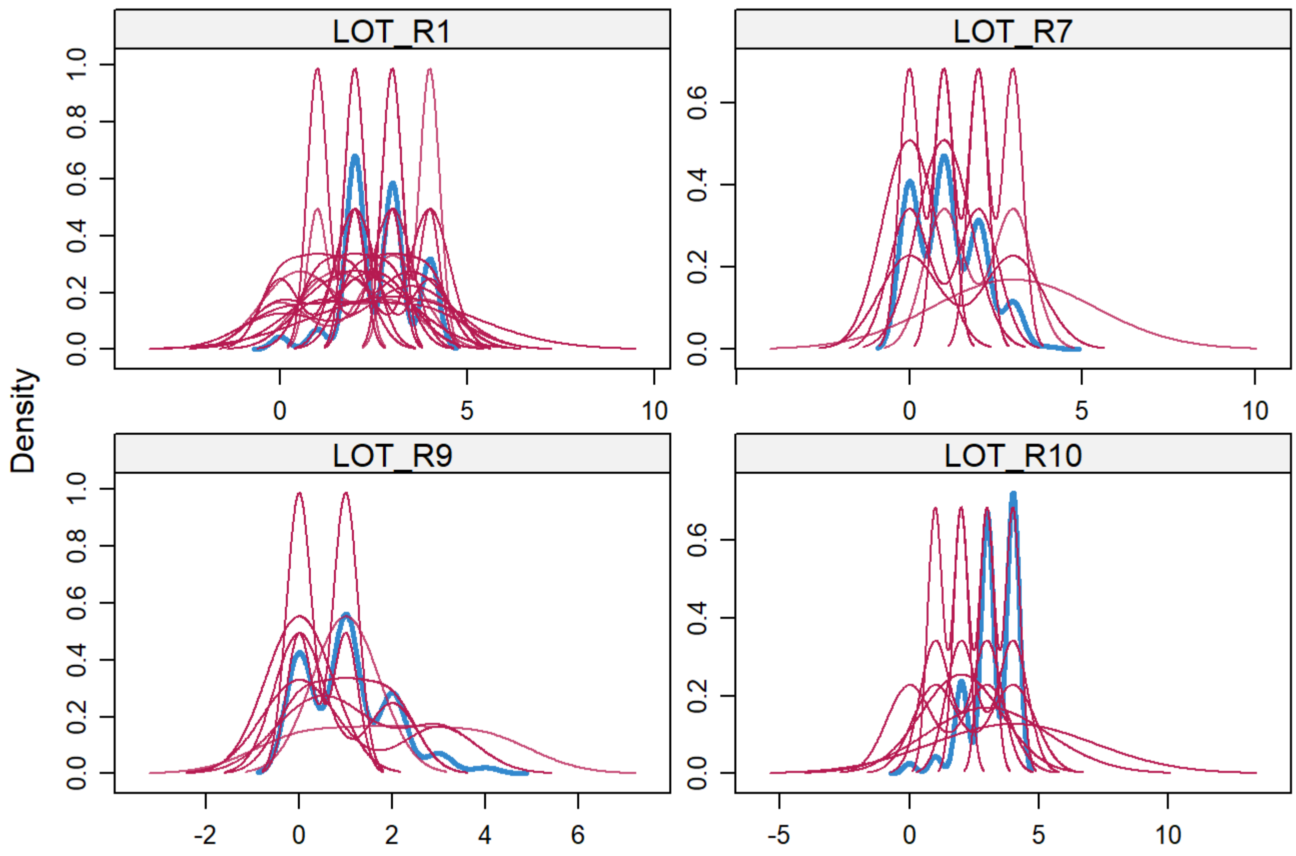


```
densityplot(imp_run, ~ HADS6+HADS8)
```

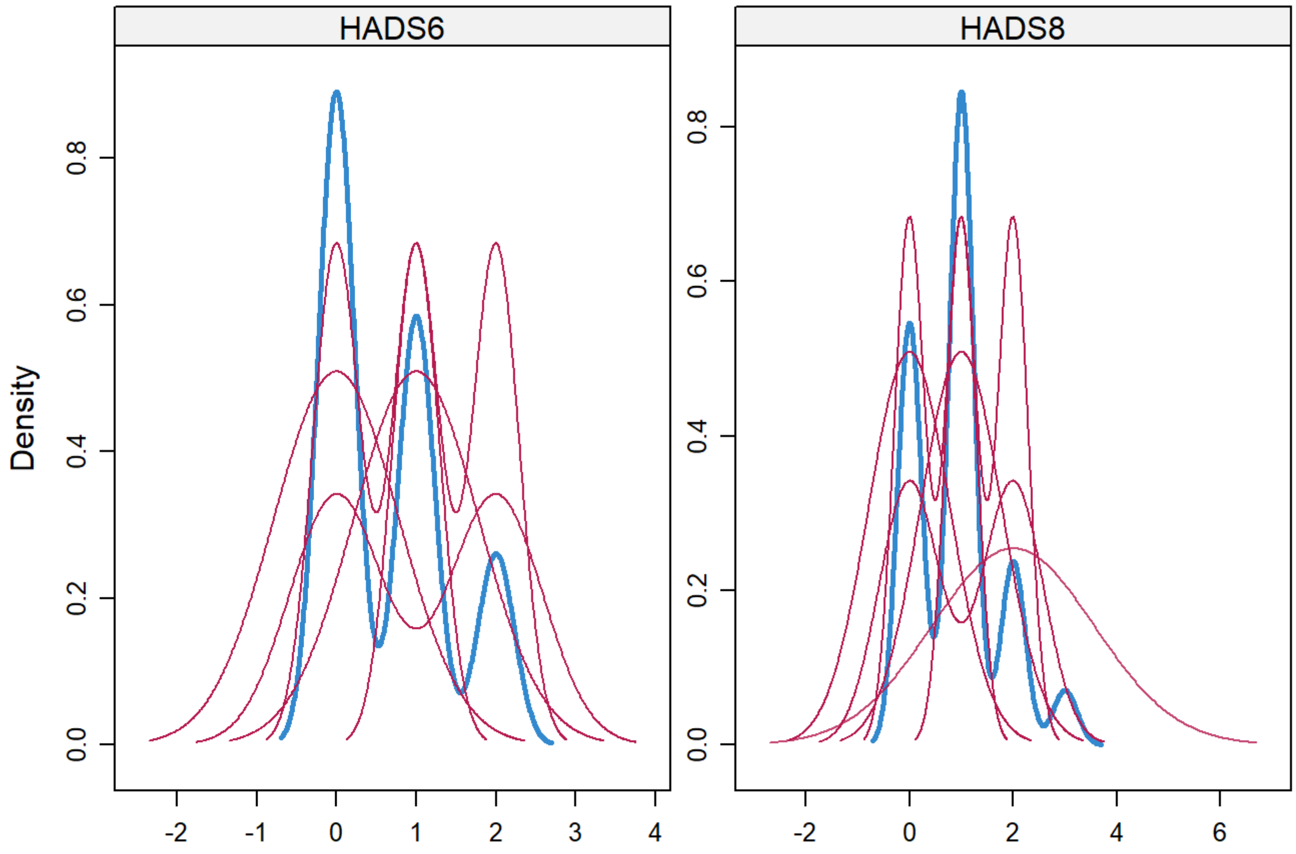

```
densityplot(imp_run, ~ IEQ1+IEQ2+IEQ3+IEQ4+IEQ5)
```

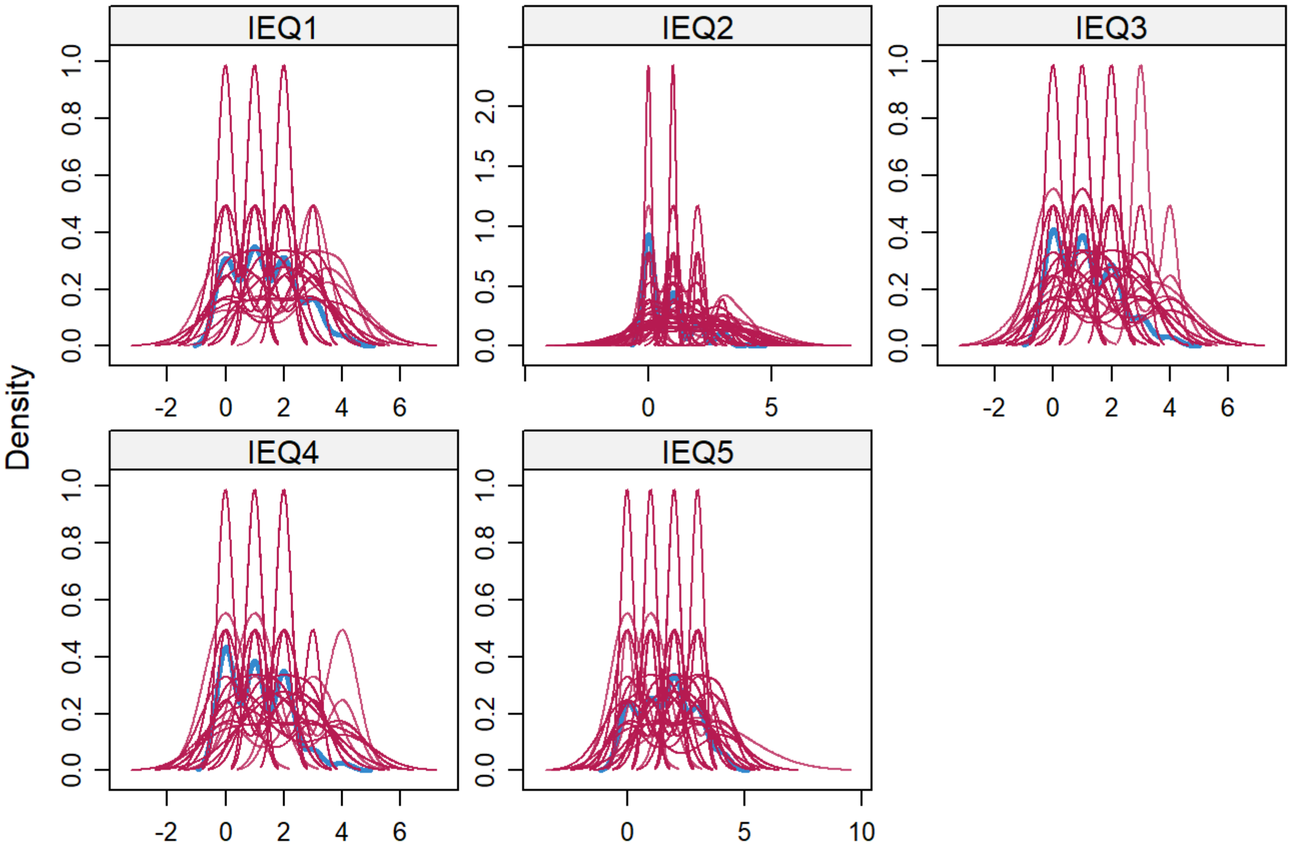


```
densityplot(imp_run, ~ SocSup1+SocSup3+SocSup5+SocSup6)
```

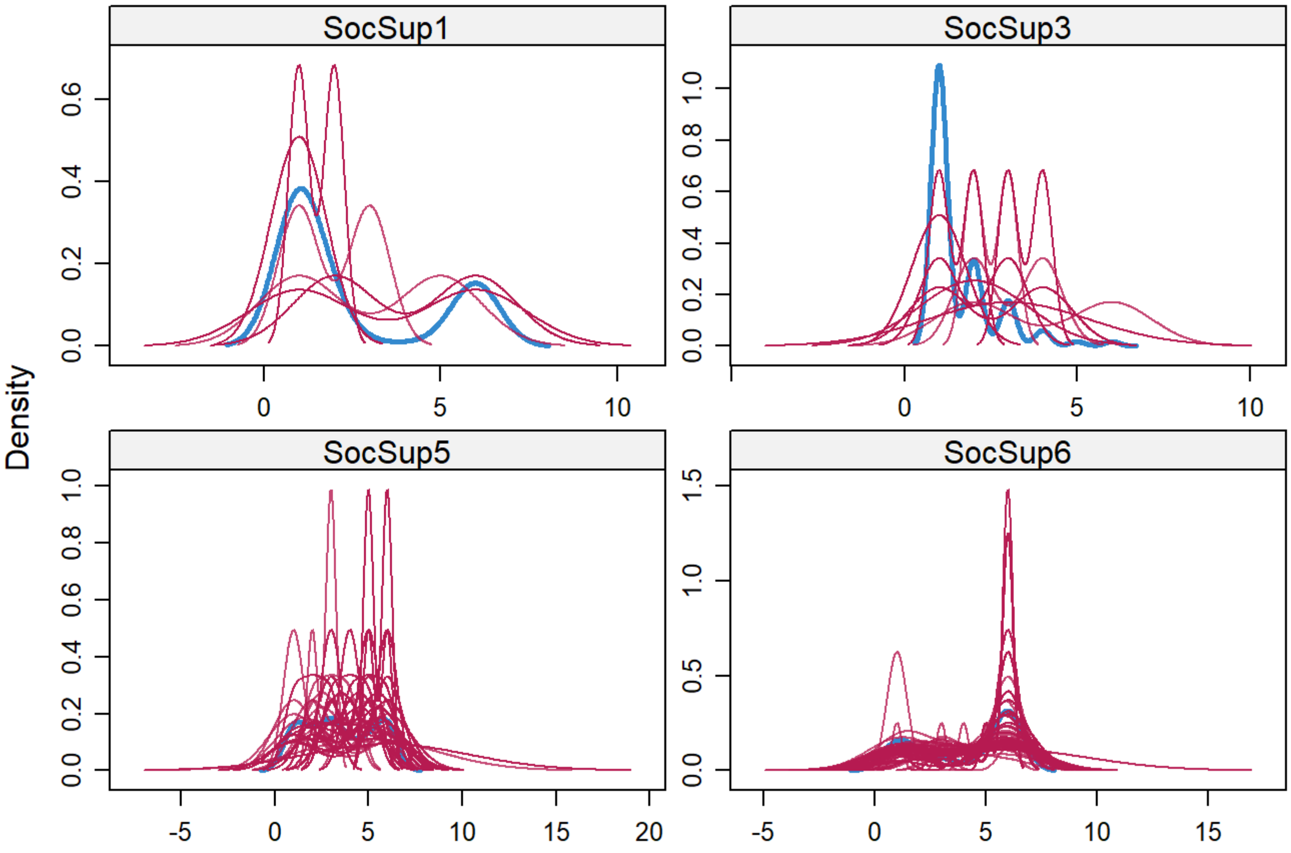


```
densityplot(imp_run, ~ PainExpectation)
```

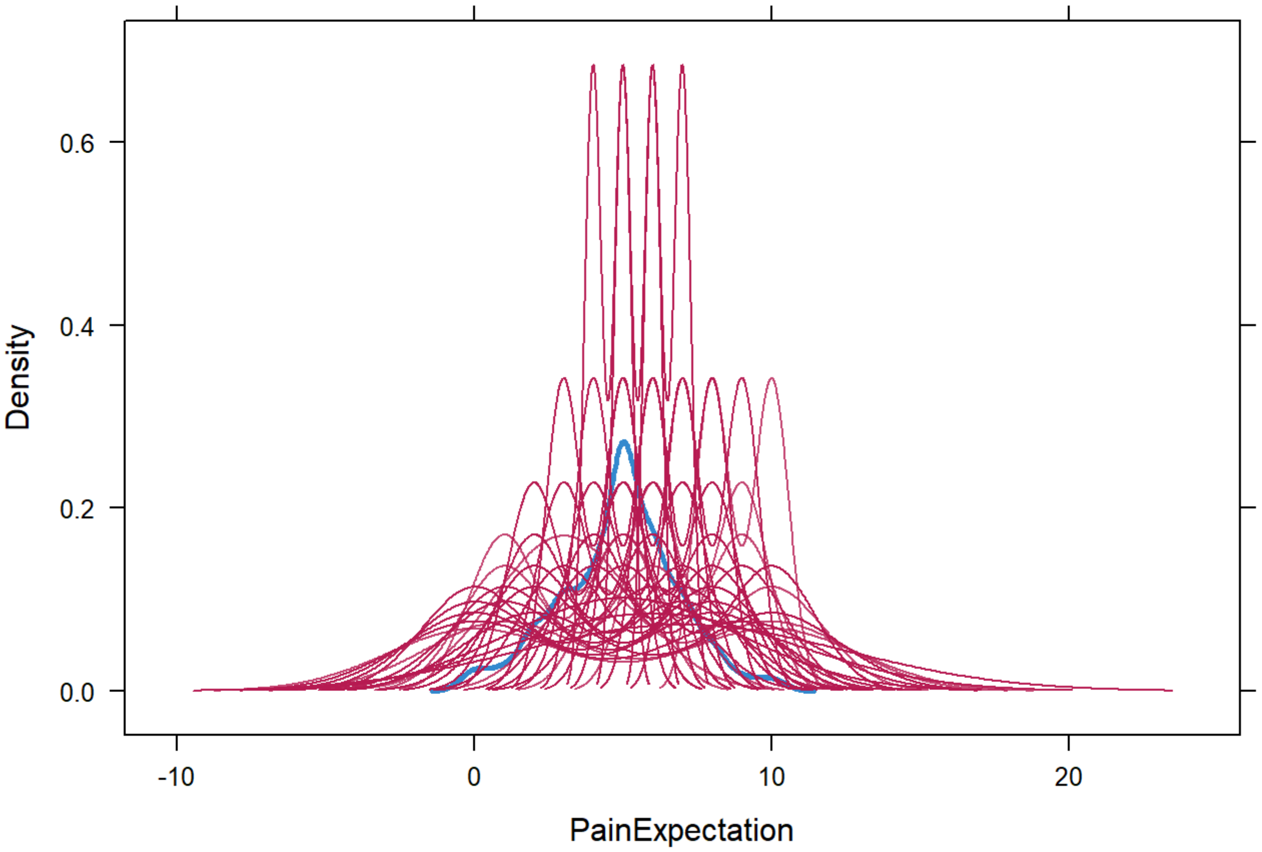

Conversion to long data

The imputation was converted to long format - including the original data with missing values.

```
imp_long <- data.frame(complete(imp_run, include = TRUE, action = 'long'))
```

Calculating total scores

Total scores were re-calculated after imputation. - Reverse codes were calculated using absolute value of non-reverse minus maximal score on the questionnaire scale - Total scores were calculated according to priorly published questionnaire scoring keys - Grouping variables were created where applicable

```
#Reverse coding of items 3, 7 and 9 of LOT-R
imp_long$LOTR_3Rev <- abs(imp_long$LOT_R3 - 4)
imp_long$LOTR_7Rev <- abs(imp_long$LOT_R7 - 4)
imp_long$LOTR_9Rev <- abs(imp_long$LOT_R9 - 4)

# Creating total scores of questionnaires
imp_long$SFQ <- rowSums(imp_long[c(paste0('SFQ', 1:8))])
imp_long$SFQSImp <- rowSums(imp_long[c(paste0('SFQ', c(1,2,3,4)))])
imp_long$SFQLImp <- rowSums(imp_long[c(paste0('SFQ', c(5,6,7,8)))])

imp_long$IEQ <- rowSums(imp_long[c(paste0('IEQ', 1:5))])

imp_long$HADSAnxiety <- rowSums(imp_long[c(paste0('HADS',c(1,3,5,7,9,11,13)))])
imp_long$HADSDepression <- rowSums(imp_long[c(paste0('HADS',c(2,4,6,8,10,12,14)))])

imp_long$LOT_R <- rowSums(imp_long[c(paste0('LOT_R', c(1,4,10)),'LOTR_3Rev','LOTR_7Re
v', 'LOTR_9Rev')])

imp_long$SocSupR1 <- dplyr::recode(imp_long$SocSup1, `1`=6L,`2`=5L,`3`=4L,`4`=3L,`5`=
2L,`6`=1L)
imp_long$SocSupR2 <- dplyr::recode(imp_long$SocSup2, `1`=6L,`2`=5L,`3`=4L,`4`=3L,`5`=
2L,`6`=1L)
imp_long$SocSupR3 <- dplyr::recode(imp_long$SocSup3, `1`=6L,`2`=5L,`3`=4L,`4`=3L,`5`=
2L,`6`=1L)
imp_long$SocSupR4 <- dplyr::recode(imp_long$SocSup4, `1`=6L,`2`=5L,`3`=4L,`4`=3L,`5`=
2L,`6`=1L)
imp_long$SocSupR5 <- dplyr::recode(imp_long$SocSup5, `1`=6L,`2`=5L,`3`=4L,`4`=3L,`5`=
2L,`6`=1L)
imp_long$SocSupR6 <- dplyr::recode(imp_long$SocSup6, `1`=6L,`2`=5L,`3`=4L,`4`=3L,`5`=
2L,`6`=1L)

imp_long$SocialSupport <- rowSums(imp_long[c(paste0('SocSupR', 1:6))])

#Grouping variable SFQGroup = low, moderate, high fear
imp_long$SFQGroup[imp_long$SFQ <=13] <- 1
imp_long$SFQGroup[imp_long$SFQ >= 14 & imp_long$SFQ <= 35] <- 2
imp_long$SFQGroup[imp_long$SFQ >=36] <- 3

# Mastectomy or breast reconstruction
imp_long$Surgery[imp_long$Mastectomy == 1 | imp_long$BreastReconstruction == 1] <- 2
imp_long$Surgery[is.na(imp_long$Surgery)] <- 1

# Any type of breast conserving surgery
imp_long$BCT[imp_long$BCTOnco == 1 | imp_long$BCTOnly == 1] <- 2
imp_long$BCT[is.na(imp_long$BCT)] <- 1

# Grouped surgery / BCT
imp_long$SurgTreat[imp_long$Surgery == 0 & imp_long$BCT == 0] <- 0
imp_long$SurgTreat[imp_long$Surgery == 1 & imp_long$BCT == 0] <- 1
imp_long$SurgTreat[imp_long$Surgery == 0 & imp_long$BCT == 1] <- 2
imp_long$SurgTreat[imp_long$Surgery == 1 & imp_long$BCT == 1] <- 3

# Chronic vs Sporadic pain
imp_long$NRS_pain_sum <- rowSums(sapply(imp_long[c(24:28)], as.numeric)-1, na.rm = T)
imp_long$NRS_painType_sum <- rowSums(sapply(imp_long[c(29:33)], as.numeric)-1, na.rm
```

```
= T)

imp_long$ChronicPain[imp_long$NRS_pain_sum >= 1 & imp_long$NRS_painType_sum >= 1] <-
2
imp_long$ChronicPain[is.na(imp_long$ChronicPain)] <- 1
imp_long$SporadicPain[imp_long$NRS_pain_sum >= 1 & imp_long$NRS_painType_sum == 0] <-
2
imp_long$SporadicPain[is.na(imp_long$SporadicPain)] <- 1
```

Converting to MIDS format

Long format imputation data was then transformed into MIDS format, necessary for several analyses.

```
imp_mids <- as.mids(imp_long, where = NULL, .imp = ".imp", .id = ".id")
```

Dataset with imputation only

Finally, a dataset including all imputations but excluding the initial data was generated.

```
imp_long_excl <- imp_long[imp_long$.imp != 0,]
imp_long_excl$SFQGroup <- as.factor(imp_long_excl$SFQGroup)
```

Imputed analysis

Descriptives

Continuous variables

Descriptive measures, including mean, sd, quantiles, IQR, min, max, skewness and kurtosis were obtained for continuous variables including questionnaire total scores using a for loop. Within the for loop, in each iteration, these measures are calculated and pooled afterwards.

```
Quest <- c('SFQ', 'Age', 'IEQ',  'HADSAnxiety', 'HADSDepression','LOT_R', 'SocialSupp
ort', 'PriorSurg','PainExpectation')
Descriptives_Questionnaires <- matrix(nrow = 1, ncol = 11)

for (i in Quest){
  imp_long_excl[[i]] <- as.numeric(imp_long_excl[[i]])
  Questitems <- c(with(imp_long_excl,
                       by(imp_long_excl, .imp, function(x)
                         c(length((x[[i]])),mean(x[[i]]),sd(x[[i]]),
                           quantile(x[[i]], c(.50, .25, .75)),IQR(x[[i]]),
                           min(x[[i]]),max(x[[i]]),skewness(x[[i]], type=2),kurtosis
(x[[i]])))))
  Questitem<- Reduce("+",Questitems)/length(Questitems)
  Descriptives_Questionnaires <- rbind(Descriptives_Questionnaires,
                                       round(Questitem, digits = 2))}
Descriptives_Questionnaires <- data.frame(Descriptives_Questionnaires)
Descriptives_Questionnaires <- Descriptives_Questionnaires[-c(1),]
colnames(Descriptives_Questionnaires) <- c('N', 'Mean', 'SD','Median','25th Percentil
e', '75th Percentile','IQR','Min', 'Max', 'Skewness','Kurtosis')
rownames(Descriptives_Questionnaires) <- Quest
```

Categorical variables

Descriptive measures, including n and percentage were obtained for categorical variables using a for loop. Again, within the for loop, in each iteration, these measures are calculated and pooled afterwards. Factors with more then 2 levels were also incorporated by expanding the quantification with 'x[[i]]==' values bigger then 1'.

```
Questcat <- c('MaritalSt2G', 'Children', 'Educ2G','OccupationalStatus','PreviousCance
r','Psychiatric', 'PsychDrugs','PriorBreastSurg','Diagnosis','Neoadjuvant','Mastectom
y', 'BreastReconstruction', 'BCT', 'SN', 'ALND','Hospital','SporadicPain', 'ChronicPa
in')

Descriptives_Questionnaires <- matrix(nrow = 1, ncol = 13)
for (i in Questcat){
  imp_long_excl[[i]] <- as.factor(imp_long_excl[[i]])
  Questitems <- c(with(imp_long_excl,
                       by(imp_long_excl, .imp, function(x)
                         c(length((!is.na(x[[i]]))),
                           length((x[[i]][x[[i]]==0])),
                           length((x[[i]][x[[i]]==1])),
                           length((x[[i]][x[[i]]==2])),
                           length((x[[i]][x[[i]]==3])),
                           length((x[[i]][x[[i]]==4])),
                           length((x[[i]][x[[i]]==5])),
                           round(length((x[[i]][x[[i]]==0]))/length(!is.na(x[[i]]))*1
00,2),
                           round(length((x[[i]][x[[i]]==1]))/length(!is.na(x[[i]]))*1
00,2),
                           round(length((x[[i]][x[[i]]==2]))/length(!is.na(x[[i]]))*1
00,2),
                           round(length((x[[i]][x[[i]]==3]))/length(!is.na(x[[i]]))*1
00,2),
                           round(length((x[[i]][x[[i]]==4]))/length(!is.na(x[[i]]))*1
00,2),
                           round(length((x[[i]][x[[i]]==5]))/length(!is.na(x[[i]]))*1
00,2)
                         ))))
  Questitem<- Reduce("+",Questitems)/length(Questitems)
  Descriptives_Questionnaires <- rbind(Descriptives_Questionnaires,
                                       round(Questitem, digits = 2))}
Descriptives_Questionnaires <- data.frame(Descriptives_Questionnaires)
Descriptives_Questionnaires <- Descriptives_Questionnaires[-c(1),]
colnames(Descriptives_Questionnaires) <- c('N','n0','n1','n2','n3','n4','n5', '%0','%
1', '%2', '%3', '%4', '%5')
rownames(Descriptives_Questionnaires) <- Questcat
```

SFQ subscales

Short-term and long-term fear of surgery (SFQ subscales) in the same way as done above.

```
# Descriptives - Short- and long-term fears - pooled
SFQSL <- c('SFQSImp', 'SFQLImp')
Descriptives_SFQSL <- matrix(nrow = 1, ncol = 11)
for (i in SFQSL){
  imp_long_excl[[i]] <- as.numeric(imp_long_excl[[i]])
  QuestSFQSL <- c(with(imp_long_excl, by(imp_long_excl, .imp,function(x)
    c(length(x[[i]]),mean(x[[i]]),sd(x[[i]]),quantile(x[[i]]),IQR(x[[i]]),skewness(x
[[i]]), kurtosis(x[[i]])))))
  Quest_SFQSL<- Reduce("+",QuestSFQSL)/length(QuestSFQSL)
  Descriptives_SFQSL <- rbind(Descriptives_SFQSL, round(Quest_SFQSL, digits = 2))
}

Descriptives_SFQSL <- data.frame(Descriptives_SFQSL)
Descriptives_SFQSL <- Descriptives_SFQSL[-c(1),]
colnames(Descriptives_SFQSL) <- c('N', 'Mean','SD','Min', '25th Percentile','Media
n','75th Percentile', 'Max','IQR','Skewness', 'Kurtosis')
rownames(Descriptives_SFQSL) <- SFQSL
```

SFQ items

Lastly, SFQ items were analysed seperately and restructured in descending order in the dataframe.

```
SFQitems <- c('SFQ1', 'SFQ2', 'SFQ3', 'SFQ4', 'SFQ5', 'SFQ6', 'SFQ7', 'SFQ8')
Descriptives_SFQitems <- matrix(nrow = 1, ncol = 11)
for (i in SFQitems){
  QuestSFQitems <- c(with(imp_long, by(imp_long, .imp, function(x)
    c(length(x[[i]]),mean(x[[i]],na.rm=T),sd(x[[i]],na.rm=T),
      quantile(!is.na(x[[i]]), c(.50, .25, .75)),IQR(x[[i]],na.rm=T),
      min(x[[i]],na.rm=T),max(x[[i]],na.rm=T),skewness(x[[i]], type=2),
      kurtosis(x[[i]], type=2)))))
  Quest_SFQitems<- Reduce("+",QuestSFQitems)/length(QuestSFQitems)
  Descriptives_SFQitems <- rbind(Descriptives_SFQitems,
                                 round(Quest_SFQitems, digits = 2))
}

Descriptives_SFQitems <- data.frame(Descriptives_SFQitems)
Descriptives_SFQitems <- Descriptives_SFQitems[-c(1),]
colnames(Descriptives_SFQitems) <- c('N', 'Mean', 'SD','Median','25th Percentile', '7
5th Percentile','IQR','Min', 'Max', 'Skewness', 'Kurtosis')
rownames(Descriptives_SFQitems) <- c('Surgery', 'Anesthesia','Postoperative pain','Si
de effects','Fear of health-deterioration', 'Failed surgery','Incomplete recovery','L
ong duration of rehabilitation')

Descriptives_SFQitems <- Descriptives_SFQitems[order(-Descriptives_SFQitems$Mean),]
```

T-test SFQ subscales

A paired samples t-test was employed to test whether there was a significant difference between short-term and long-term fear of surgery (again pooled afterwards).

```
SFQttest <- with(imp_long, by(imp_long, .imp, function(x)
  c(t.test(x$SFQSImp, x$SFQLImp, paired = TRUE, alternative = 'greater'))))

pvalues <- c()
tvalues <- c()
dfvalues <- c()
for (i in names(SFQttest)){
  pvalue <- SFQttest[[i]]$p.value
  pvalues <- c(pvalues, pvalue)
  tvalue <- SFQttest[[i]]$statistic
  tvalues <- c(tvalues, tvalue)
  dfvalue <- SFQttest[[i]]$parameter
  dfvalues <- c(dfvalues, dfvalue)
}
paste0('T(', round(Reduce("+",dfvalues)/length(dfvalues),2), ')=',round(Reduce("+",tv
alues)/length(tvalues),3), ', p = ', round(Reduce("+",pvalues)/length(pvalues),3))
```

Violin plots

SFQ total

Calculation of mean SFQ total scores:

```
# Calculations of mean scores
SFQmean <- with(imp_long_excl, by(imp_long_excl, .imp, function(x)
  c(mean(x$SFQ))))
SFQmeanP <- Reduce("+",SFQmean)/length(SFQmean)
```

Thereafter, a violin plot was coded to obtain a visual representation of the distribution of total scores of the SFQ questionnaire.

```r
p <- ggplot() +
  geom_violin(data = imp_long_excl,aes(x ='',y = SFQ))
p_build <- ggplot2::ggplot_build(p)$data[[1]]
p_build <- transform(p_build,
                     xminv = x - violinwidth * (x - xmin),
                     xmaxv = x + violinwidth * (xmax - x))
p_build <- rbind(plyr::arrange(transform(p_build, x = xminv), y),
                 plyr::arrange(transform(p_build, x = xmaxv), -y))
colors <- c("#808080", "#E3D4B1", "#F1D7D8")
#Add our fill variable
p_build$FearGroup[p_build$y <= 14] <- 'Low'
p_build$FearGroup[p_build$y >= 36] <- 'High'
p_build$FearGroup[is.na(p_build$FearGroup)] <- 'Moderate'
p_build$FearGroup <- factor(p_build$FearGroup,
                            levels = c("Low", "Moderate", "High"))
ggplot() +
  geom_polygon(data = p_build,
             aes(x = x,y = y,fill = FearGroup))+
  stat_boxplot(geom ='errorbar', width = 0.1) +
  geom_boxplot(data = imp_long, aes(x = 1,y = SFQ), width=0.1)+
  theme(panel.grid.major = element_blank(), panel.grid.minor = element_blank(),
        panel.background = element_blank(),
        axis.line = element_line(colour = "black"), legend.position="bottom",
        axis.ticks.x = element_blank(),
        axis.text.x = element_blank(),
        axis.text.y = element_text(colour='black')) +
  labs(x="",y= "SFQ Total Score") +
  annotate(geom="text", x=1, y=26.42+3,
         label= paste0("M= ", round(SFQmeanP,digits = 2)))+
  stat_summary(fun = mean, geom = "errorbar", aes(ymax = ..y.., ymin = ..y..),
             width = .1, color="blue") +
  scale_fill_manual(values = setNames(colors, levels(p_build$FearGroup)))
```

SFQ subscales

The same was done for the subscales of the SFQ questionnaire - short- and long-term.

```r
SFQsub <- data.frame('SFQS' = imp_long$SFQSImp, 'SFQL' = imp_long$SFQLImp)
SFQsubBP <- melt(SFQsub)
ggplot(SFQsubBP, aes(x = variable, y= value))+
  geom_violin(fill='#CBCBCB', alpha=0.7,adjust=1.5) +
  geom_boxplot(width=0.1, fill='#FFFFFF') +
  theme(panel.grid.major = element_blank(), panel.grid.minor = element_blank(),
        panel.background = element_blank(), text = element_text(size=12),
        axis.line = element_line(colour = "black"), legend.position="bottom",
        axis.text = element_text(colour='black')) +
  labs(x= "SFQ Subscales", y= "Score per subscale")
```

SFQ single items

Lastly, the distribution of scores on all single items was visualized.

```
SFQsub2 <- data.frame('Surgery' = imp_long$SFQ1,
                      'Health deterioration' = imp_long$SFQ5,
                      'Postoperative pain' = imp_long$SFQ3,
                      'Side effects' = imp_long$SFQ4,
                      'Long rehabilitation' = imp_long$SFQ8,
                      'Failed surgery' = imp_long$SFQ6,
                      'Anesthesia' = imp_long$SFQ2,
                      'Incomplete recovery' = imp_long$SFQ7)
SFQsubBP2 <- melt(SFQsub2)
ggplot(SFQsubBP2, aes(x = variable, y= value))+
  geom_violin(fill='#CBCBCB',alpha=0.7,adjust=1.5) +
  geom_boxplot(width=0.1, fill='#FFFFFF') +
  theme(panel.grid.major = element_blank(), panel.grid.minor = element_blank(),
        panel.background = element_blank(),
        axis.line = element_line(colour = "black"), legend.position="bottom",
        axis.text.y = element_text(colour='black')) +
  theme(legend.position="bottom") +
  labs(x= "SFQ items", y= "Score per item") +
  theme(axis.text.x = element_text(angle = 80, vjust= 1, hjust = 1, size = 18), text
= element_text(size=18))+
  scale_x_discrete(labels=c("Surgery" = "Surgery",
                            "Health.deterioration" = "Health deterioration",
                            "Postoperative.pain" = "Postoperative pain",
                            "Side.effects" = "Side effects",
                            "Long.rehabilitation" = "Long rehabilitation",
                            "Failed.surgery" = "Failed surgery",
                            "Anesthesia" = "Anesthesia",
                            "Incomplete.recovery"="Incomplete recovery"))
```

ANOVA between SFQ groups

Continuous descriptives

Descriptive analyses of continuous variables per fear group.

```r
numdescr <- data.frame(matrix(ncol=3))
numlist <- c('Age', 'PriorSurg', 'PainExpectation', 'HADSAnxiety','HADSDepression',
'IEQ', 'LOT_R', 'SocialSupport')
for (variable in numlist){
  numitem <- c()
  for (i in c(1,2,3)){
    fit <- with(imp_long_excl[imp_long_excl$SFQGroup == i,],
                by(imp_long_excl[imp_long_excl$SFQGroup == i,], .imp,
                   function(x) c(mean(x[[variable]]),sd(x[[variable]]))))
    item <- round(Reduce("+",fit)/length(fit),digits = 2)
    item <- paste0(item[1], ' (', item[2], ')')
    numitem <- cbind(numitem, item)
  }
  colnames(numdescr) <- colnames(numitem)
  numdescr <- rbind(numdescr, numitem)
}
numdescr <- numdescr[-1,]
numdescr <- data.frame(numdescr)
rownames(numdescr)<-numlist
colnames(numdescr)<-c('low','mod','high')
```

Categorical descriptives

The same was done for categorical variables.

```
CatDesItem <- c('MaritalSt2G', 'Children','Educ2G', 'OccupationalStatus','PreviousCan
cer', 'Psychiatric', 'PsychDrugs','PriorBreastSurg', 'Neoadjuvant', 'SN', 'ALND', 'Ho
spital','SporadicPain','ChronicPain','Surgery','BCT')
CatDesDF <- data.frame(matrix(ncol = 3))
for (i in CatDesItem){
  CatDesRow <- c()
  for (group in c(1,2,3)){
    Items <- c(with(imp_long_excl[imp_long_excl$SFQGroup == group,],
                    by(imp_long_excl[imp_long_excl$SFQGroup == group,],
                       .imp, function(x)
                         c(length(x[[i]][x[[i]]==1]),
                           (length(x[[i]][x[[i]]==1])/length(x[[i]])*100)))))
    Cat_Des<- Reduce("+",Items)/length(Items)
    Cat_Des<- paste0(round(Cat_Des[1], digits = 0), ' (',
                     round(Cat_Des[2],digits = 2), '%)')
    CatDesRow <- cbind(CatDesRow, Cat_Des)
  }
  colnames(CatDesDF) <- colnames(CatDesRow)
  CatDesDF <- rbind(CatDesDF, CatDesRow, names(c(1,2,3)))
}
CatDesDF <- CatDesDF[-1,]
colnames(CatDesDF) <- c('LOW- N(%)','MOD- N(%)','HIGH- N(%)')
rownames(CatDesDF) <- CatDesItem

CatDesDF2 <- data.frame(matrix(ncol = 3))
for (i in c('Diagnosis')){
  for (cat in c(1:5)){
  CatDesRow <- c()
    for (group in c(1,2,3)){
      Items <- c(with(imp_long_excl[imp_long_excl$SFQGroup == group,],
                      by(imp_long_excl[imp_long_excl$SFQGroup == group,],
                         .imp, function(x)
                           c(length(x[[i]][x[[i]]==cat]),
                             (length(x[[i]][x[[i]]==cat])/length(x[[i]])*100)))))
      Cat_Des<- Reduce("+",Items)/length(Items)
      Cat_Des<- paste0(round(Cat_Des[1], digits = 0), ' (',
                       round(Cat_Des[2],digits = 2), '%)')
      CatDesRow <- cbind(CatDesRow, Cat_Des)
  }
  colnames(CatDesDF2) <- colnames(CatDesRow)
  CatDesDF2 <- rbind(CatDesDF2, CatDesRow, names(c(1,2,3)))}
}
CatDesDF2 <- CatDesDF2[-1,]
colnames(CatDesDF2) <- c('LOW- N(%)','MOD- N(%)','HIGH- N(%)')
rownames(CatDesDF2) <- c('primaryNonInvasiveBC','primaryInvasiveBC',
                        'primaryBC+metastasis','RecurrentBC','RecurrentBC+metastasi
s')
```

ANOVA

Preparation for ANOVA - transformation of variables to numeric.

```r
# Set variable classes
imp_long_excl$SFQGroup <- as.factor(imp_long_excl$SFQGroup)
for (i in c('Children', 'PreviousCancer', 'Psychiatric', 'PsychDrugs',
            'PriorSurg','Neoadjuvant', 'Hospital', 'Mastectomy',
            'BreastReconstruction','BCT', 'SN', 'ALND', 'ChronicPain',
            'SporadicPain','Age', 'HADSAnxiety',
            'HADSDepression','IEQ', 'LOT_R', 'SocialSupport',
            'PainExpectation')){
  imp_long_excl[[i]] <- as.numeric(imp_long_excl[[i]])
}
```

A for loop was setup to perform all ANOVA’s and post-hoc tests. Firstly, a Levene’s test is used to determine if unqual variance can be assumed or not. In cases equal variance could not be assumed (Levene’s test significant) a welch F test in combination with Games-Howell post hoc test was used. The Games-Howell post hoc was incorporated to the final dataframe outside of the for loop due to unsuitable function setup to use in for loop. In cases where equal variances could be assumed (Levene’s test not significant), a ANOVA F test was used in combination with Tukey post-hoc.

```
ANOVA_list <- c('Age','PainExpectation', 'HADSAnxiety',
                'HADSDepression','IEQ', 'LOT_R', 'SocialSupport', 'PriorSurg')

anovaOverview<- c()
for (x in ANOVA_list){
  LevT <- c()
  for (i in c(1:2)){
    dataANOVA <- imp_long_excl[imp_long_excl$.imp == i,]
    fit = leveneTest(dataANOVA[[x]] ~ dataANOVA$SFQGroup)
    LevT <- rbind(LevT,fit$`Pr(>F)`)}

  # Levene's significant -> welchF + Games Howell + varequal false
  fitLong = data.frame(matrix(ncol=7))
  if (mean(LevT[,1]) < 0.05) {
    for (i in c(1:2)){
      fit <- oneway.test(dataANOVA[[x]] ~ dataANOVA$SFQGroup,
                         var.equal = FALSE)
      #PostHoc <- games_howell_test(data = dataANOVA, [[x]] ~ SFQGroup, conf.level =
0.95, detailed = FALSE)

      fitLong <- rbind(fitLong, c(fit$parameter[1], fit$parameter[2],
                                  fit$statistic, fit$p.value, NA,NA,NA
                                  #PostHoc$p.adj[1],PostHoc$p.adj[2],PostHoc$p.adj[3]
      ))}}

  # Levene's not significant -> ANOVA F + Tukey + varequal true
  if (mean(LevT[,1]) > 0.05) {
    for (i in c(1:2)){
      fit <- aov(dataANOVA[[x]] ~ dataANOVA$SFQGroup)
      ANOVA <- summary(fit)
      Tukey <- TukeyHSD(fit)
      fitLong <- rbind(fitLong, c(ANOVA[[1]][["Df"]][1], ANOVA[[1]][["Df"]][2],
                                  ANOVA[[1]][["F value"]][1],
                                  ANOVA[[1]][["Pr(>F)"]][1],
                                  Tukey$`dataANOVA$SFQGroup`[2,"p adj"],
                                  Tukey$`dataANOVA$SFQGroup`[3,"p adj"],
                                  Tukey$`dataANOVA$SFQGroup`[1,"p adj"]))}}

  fitLong <- fitLong[-1,]
  colnames(fitLong) <- c('df1', 'df2', 'F', 'p', 'G1', 'G2', 'G3')
  stat <- paste0('F(',mean(fitLong$df1), ',', mean(fitLong$df2), ')',
                 round(mean(fitLong$F),digits = 3))
  p <- mean(fitLong$p)
  PH <- cbind(round(mean(fitLong$G1),digits = 3),
              round(mean(fitLong$G2),digits = 3),
              round(mean(fitLong$G3),digits = 3))
  item <- cbind(stat,p,PH)

  anovaOverview <- rbind(anovaOverview, item)
}
anovaOverview2 <- data.frame(anovaOverview)
```

As mentioned before, Games-Howell post-hoc tests were added to the final dataframe outside of the for loop due to inability to incorporate it.

```
PostHoc <- games_howell_test(data = dataANOVA,  HADSDepression ~ SFQGroup, conf.level
= 0.95, detailed = FALSE)
anovaOverview2[4,c(3:5)] <- round(c(PostHoc$p.adj[2],PostHoc$p.adj[3],PostHoc$p.adj
[1]),3)
PostHoc <-games_howell_test(data = dataANOVA,  IEQ ~ SFQGroup, conf.level = 0.95, det
ailed = FALSE)
anovaOverview2[5,c(3:5)] <- round(c(PostHoc$p.adj[2],PostHoc$p.adj[3],PostHoc$p.adj
[1]),3)
PostHoc <-games_howell_test(data = dataANOVA,  PriorSurg ~ SFQGroup, conf.level = 0.9
5, detailed = FALSE)
anovaOverview2[8,c(3:5)] <- round(c(PostHoc$p.adj[2],PostHoc$p.adj[3],PostHoc$p.adj
[1]),3)

colnames(anovaOverview2) <- c('stat', 'p', '1vs3', '2vs3', '1vs2')
rownames(anovaOverview2) <- ANOVA_list
```

Chi-Square

Also for the chi-square analysis, variables were first classified as numeric.

```
# Changing variable class
ChiVars <- c('MaritalSt2G', 'Children', 'Educ2G', 'OccupationalStatus','PreviousCance
r', 'Psychiatric', 'PsychDrugs','PriorBreastSurg','Diagnosis','Neoadjuvant','Surger
y','BCT', 'SN','ALND','Hospital','ChronicPain', 'SporadicPain','SFQGroup')
for (i in ChiVars){
  imp_long_excl[[i]] <- as.numeric(imp_long_excl[[i]])
}
```

Chi-Square analysis was also performed in a for loop, going over each variable and each imputation seperately before pooling the results. Bonferoni post-hoc test was performed. If chi-square tests were not significant, post-hoc analyses were removed (‘NA’).

```
ChiSquares <- data.frame(matrix(ncol = 5))
Chi_var <- c('MaritalSt2G', 'Children', 'Educ2G', 'OccupationalStatus','PreviousCance
r', 'Psychiatric', 'PsychDrugs','PriorBreastSurg','Diagnosis','Neoadjuvant','Surger
y','BCT', 'SN','ALND','Hospital','ChronicPain', 'SporadicPain')

for (variable in Chi_var){
  chi_total <- c()
  chi_item_mean <- c()
  for (i in c(1:100)){
    chi_DF <- imp_long_excl[imp_long_excl$.imp == i,]
    M <- as.table(rbind(c(length(chi_DF[[variable]][chi_DF$SFQGroup == 1 & chi_DF[[va
riable]] == 1]),
                          length(chi_DF[[variable]][chi_DF$SFQGroup == 2 & chi_DF[[va
riable]] == 1]),
                          length(chi_DF[[variable]][chi_DF$SFQGroup == 3 & chi_DF[[va
riable]] == 1])),
                        c(length(chi_DF[[variable]][chi_DF$SFQGroup == 1 & chi_DF[[va
riable]] == 2]),
                          length(chi_DF[[variable]][chi_DF$SFQGroup == 2 & chi_DF[[va
riable]] == 2]),
                          length(chi_DF[[variable]][chi_DF$SFQGroup == 3 & chi_DF[[va
riable]] == 2]))))
    dimnames(M) <- list(MaritalSt = c("Y", "N"),
                        FearGroup = c("low","moderate", "high"))
    chisq <- chisq.test(M)
    chisqPH <- chisq.posthoc.test(M, method = "bonferroni")

    if (any(chisq$expected<=5) == TRUE){
      Fis_test <- fisher.test(M)
      if (Fis_test$p > 0.05){
        chi_item <- c(chisq$parameter,chisq$statistic,
                      chisq$p.value,chisqPH$mod[4],
                      chisqPH$moderate[4],chisqPH$high[4])
      }
      if (Fis_test$p <= 0.05){
        chi_item <- c(NA,NA,NA,NA,NA,NA,NA)
      }
    }
    if (any(chisq$expected<=5) == FALSE){
      chi_item <- c(chisq$parameter,chisq$statistic,
                    chisq$p.value,chisqPH$mod[4],
                    chisqPH$moderate[4],chisqPH$high[4])
    }
    chi_total <- data.frame(rbind(chi_total, chi_item))
    colnames(chi_total) <- c('df', 'statistic', 'p', 'LowP', 'ModP', 'HighP')
  }
  chi_item_mean <- c(paste0('X2(', round(mean(chi_total$df), digits = 3), ',N=195)=',
                            round(mean(chi_total$statistic),digits = 3)),
                     mean(chi_total$p),
                     mean(chi_total$LowP),mean(chi_total$ModP),mean(chi_total$HighP))

  ChiSquares <- rbind(ChiSquares, chi_item_mean)
}
ChiSquares <- ChiSquares[-1,]
rownames(ChiSquares)<-c(Chi_var)
```

```
colnames(ChiSquares)<-c('Statistic', 'p', 'LowP', 'ModP', 'HighP')

# remove all post hoc tests where ChiSquares$p > 0.05
ChiSquares[ChiSquares$p>.05,c(3:5)] <- NA
```

# P value adjust

P values were adjusted for multiple comparison using Benjamini-Hochberg correction.

```
PadjustedValues <- p.adjust(c(anovaOverview2$p,ChiSquares$p), method = "BH")

Padjusted <- data.frame(variable = c(rownames(anovaOverview2),rownames(ChiSquares)),
                        oldP = round(c(as.numeric(anovaOverview2$p),as.numeric(ChiSqu
ares$p)),3),
                        newP = c(round(PadjustedValues,3)))
Padjusted$changedSig[Padjusted$oldP <= .05 & Padjusted$newP > .05] <- 'Changed!'
```

Venn-diagrams

To visualize the co-ocurrance of anxiety, depression and pain expectation among highly feared patients two Venn diagrams were created.

```
# Create sub-dataset
DF_venn <- imp_long %>% select(.imp, PatientID, SFQGroup, HADSAnxiety,
                               HADSDepression, PainExpectation)
# Exclude original data
DF_venn <- DF_venn[DF_venn$.imp != 0,]

# Average across all imputations
DF_venn$SFQGroup <- as.numeric(DF_venn$SFQGroup)
DF_venn_mean <- DF_venn %>%
  group_by(PatientID) %>%
  summarise(HADSDepression = mean(HADSDepression), HADSAnxiety = mean(HADSAnxiety),
            SFQGroup = mean(SFQGroup), PainExpectation=mean(PainExpectation))
# Recoding into groups
DF_venn_mean$SFQGroup[DF_venn_mean$SFQGroup == 1] <- 0
DF_venn_mean$SFQGroup[DF_venn_mean$SFQGroup == 2] <- 0
DF_venn_mean$SFQGroup[DF_venn_mean$SFQGroup == 3] <- 1
DF_venn_mean$HADSAnxiety[DF_venn_mean$HADSAnxiety < 8] <- 0
DF_venn_mean$HADSAnxiety[DF_venn_mean$HADSAnxiety >= 8] <- 1
DF_venn_mean$HADSDepression[DF_venn_mean$HADSDepression < 7] <- 0
DF_venn_mean$HADSDepression[DF_venn_mean$HADSDepression >= 7] <- 1
DF_venn_mean$PainExpectation[DF_venn_mean$PainExpectation < 7] <- 0
DF_venn_mean$PainExpectation[DF_venn_mean$PainExpectation >= 7] <- 1

# High Fear + HADS Depression + HADS Anxiety
venn_names1 <- c("Despression", "Anxiety", "High Fear of Surgery")
venn1 <- plot(euler(DF_venn_mean[2:4], shape = "ellipse"), quantities = list(cex = 1.
5),
              fills = c("#D9D9D9", "#FFFFFF", "#F1D7D8"),
              labels = list(labels = venn_names1, fontsize=15))
# High Fear + HADS Anxiety + Pain Expectation
venn_names2 <- c("Anxiety", "High Fear of Surgery", "Expecting Severe Pain")
venn2 <- plot(euler(DF_venn_mean[c(3:5)], shape = "ellipse"), quantities = list(cex =
1.5),
              fills = c("#FFFFFF", "#F1D7D8", "#D9D9D9"),
              labels = list(labels = venn_names2, fontsize=15))

grid.arrange(venn1, venn2, ncol=2)
```

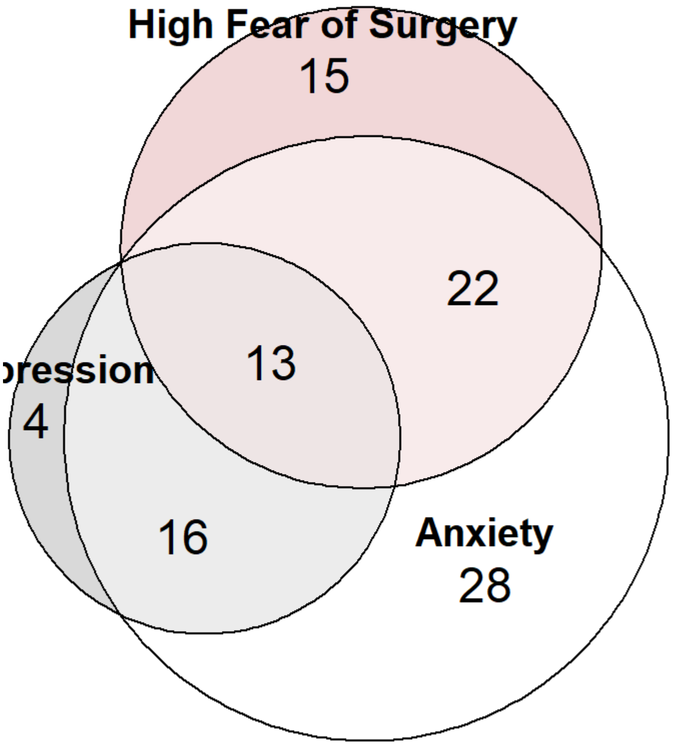

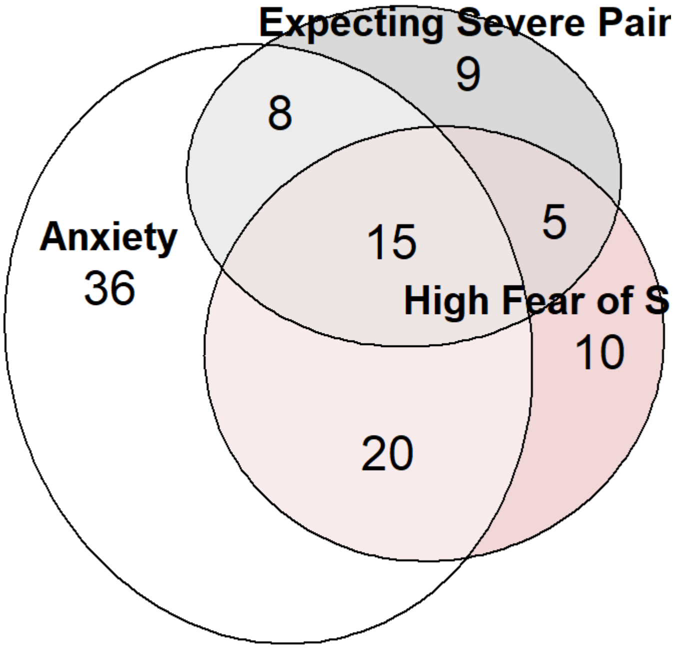

Suplementary analysis

All of the following analysis make use of code which was already used in the block of the primary analyses of the present paper. In the following section, non-imputed data was analysed to further validate correct imputation. Furthermore, two sensitivity analysis were performed. The first uses altered SFQ grouping cut-off scores. The last one compares the included participants with the excluded and non-participating but eligible patients based on general participant characteristics.

Non-imputed descriptives

Continuous variables

Total scores had to be calculated as they were not taken into the multiple imputation.

```
#Descriptives continous - not imputed
data$LOTR_3Rev <- abs(data$LOT_R3 - 4)
data$LOTR_7Rev <- abs(data$LOT_R7 - 4)
data$LOTR_9Rev <- abs(data$LOT_R9 - 4)

# Check reverse coding
# Rev_Cod_Check <- data.frame(imp_long$LOTR_3Rev,imp_long$LOT_R3)

# Creating total scores of questionnaires
data$SFQ <- rowSums(data[c(paste0('SFQ', 1:8))])
data$IEQ <- rowSums(data[c(paste0('IEQ', 1:5))])
data$HADSAnxiety <- rowSums(data[c(paste0('HADS',
                                                  c(1,3,5,7,9,11,13)))])
data$HADSDepression <- rowSums(data[c(paste0('HADS',
                                                     c(2,4,6,8,10,12,14)))])
data$LOT_R <- rowSums(data[c(paste0('LOT_R', c(1,4,10)),'LOTR_3Rev',
                                     'LOTR_7Rev', 'LOTR_9Rev')])
data$SocialSupport <- rowSums(data[c(paste0('SocSup', 1:6))])
data$SocialSupport <- abs(data$SocialSupport-36) # reverse coding
```

Continuous variable descriptive for loop.

```
DescrCON <- c('SFQ', 'Age', 'IEQ',  'HADSAnxiety', 'HADSDepression',
              'LOT_R', 'SocialSupport', 'PriorSurg','PainExpectation')
Descriptives_CON <- matrix(nrow = 1, ncol = 11)
for (i in DescrCON){
  CONitems <- c(sum(!is.na(data[[i]])), mean(data[[i]], na.rm = TRUE), sd(data[[i]],
na.rm = TRUE),
                quantile(data[[i]], c(.50, .25, .75), na.rm = TRUE),
                IQR(data[[i]], na.rm = TRUE),  min(data[[i]], na.rm = TRUE), max(data
[[i]], na.rm = TRUE),
                skewness(data[[i]], type=2, na.rm = TRUE),
                kurtosis(data[[i]], type=2, na.rm = TRUE)
  )
  Descriptives_CON <- rbind(Descriptives_CON, round(CONitems, digits = 2))
}
Descriptives_CON <- data.frame(Descriptives_CON)
Descriptives_CON <- Descriptives_CON[-c(1),]
colnames(Descriptives_CON) <- c('N', 'Mean', 'SD','Median', '25th Percentile', '75th
Percentile',
                                'IQR','Min', 'Max', 'Skewness', 'Kurtosis' )
rownames(Descriptives_CON) <- DescrCON
```

Categorical variables

Total scores had to be calculated as they were not taken into the multiple imputation.

```
data$NRS_pain_sum <- rowSums(sapply(data[c(24:28)], as.numeric)-1, na.rm = T)
data$NRS_painType_sum <- rowSums(sapply(data[c(29:33)], as.numeric)-1, na.rm = T)

data$ChronicPain[data$NRS_pain_sum >= 1 & data$NRS_painType_sum >= 1] <- 2
data$ChronicPain[is.na(data$ChronicPain)] <- 1
data$SporadicPain[data$NRS_pain_sum >= 1 & data$NRS_painType_sum == 0] <- 2
data$SporadicPain[is.na(data$SporadicPain)] <- 1

data$Surgery[data$Mastectomy == 1 | data$BreastReconstruction == 1] <- 2
data$Surgery[is.na(data$Surgery)] <- 1
data$BCT[data$BCTOnco == 1 | data$BCTOnly == 1] <- 2
data$BCT[is.na(data$BCT)] <- 1
```

Continuous variable descriptive for loop.

```
#Descriptives categorical - not imputed
DescrCAT <- c('MaritalSt2G', 'Children', 'Educ2G',
              'OccupationalStatus','PreviousCancer',
              'Psychiatric', 'PsychDrugs',
              'PriorBreastSurg','Diagnosis','Neoadjuvant',
              'Mastectomy', 'BreastReconstruction', 'BCT', 'SN', 'ALND',
              'Hospital',
              'SporadicPain', 'ChronicPain')
DescrCAT1 <- c('Diagnosis')
Descriptives_CAT <- matrix(nrow = 1, ncol = 2)
for (i in DescrCAT){
  n_total <- sum(!is.na(data[[i]]))
  One_total <- length((data[[i]][data[[i]]==1]))
  if(i=='MaritalSt2G'|i=='Educ2G'){
    One_total <- length((data[[i]][data[[i]]==2]))
  }
  DescrVar <- c(n_total, paste0(One_total,' (', round((One_total/n_total*100),digits
= 2), '%)'))

  Descriptives_CAT <- rbind(Descriptives_CAT,DescrVar)
}
for (i in DescrCAT1){
  n_total <- sum(!is.na(data[[i]]))
  One_total <- length((data[[i]][data[[i]]==1]))
  Two_total <- length((data[[i]][data[[i]]==2]))
  Three_total <- length((data[[i]][data[[i]]==3]))
  Four_total <- length((data[[i]][data[[i]]==4]))
  Five_total <- length((data[[i]][data[[i]]==5]))
  DescrVar1 <- c(n_total, paste0(One_total,' (', round((One_total/n_total*100),digits
= 2), '%)'))
  DescrVar2 <- c(n_total, paste0(Two_total,' (', round((Two_total/n_total*100),digits
= 2), '%)'))
  DescrVar3 <- c(n_total, paste0(Three_total,' (', round((Three_total/n_total*100),di
gits = 2), '%)'))
  DescrVar4 <- c(n_total,paste0(Four_total,' (', round((Four_total/n_total*100),digit
s = 2), '%)'))
  DescrVar5 <- c(n_total,paste0(Five_total,' (', round((Five_total/n_total*100),digit
s = 2), '%)'))
  Descriptives_CAT <- rbind(Descriptives_CAT,DescrVar1, DescrVar2, DescrVar3, DescrVa
r4, DescrVar5)
}
Descriptives_CAT <- data.frame(Descriptives_CAT)
Descriptives_CAT <- Descriptives_CAT[-c(1),]
colnames(Descriptives_CAT) <- c('N total', 'N,(%)' )
rownames(Descriptives_CAT) <- c(DescrCAT, 'Diagnosis1','Diagnosis2','Diagnosis3',
                                'Diagnosis4','Diagnosis5')
```

Sensitivity analysis 1

Re-categorization SFQ groups based on different grouping cut-off scores

```
imp_long_excl$SFQGroupSens <- 3
imp_long_excl$SFQGroupSens[imp_long_excl$SFQ < 44] <- 2
imp_long_excl$SFQGroupSens[imp_long_excl$SFQ < 14] <- 1
```

Continuous variable descriptive for loop.

```
numdescr <- data.frame(matrix(ncol=3))
numlist <- c('Age', 'PriorSurg', 'PainExpectation', 'HADSAnxiety',
             'HADSDepression', 'IEQ', 'LOT_R', 'SocialSupport')
for (variable in numlist){
  numitem <- c()
  for (i in c(1,2,3)){
    fit <- with(imp_long_excl[imp_long_excl$SFQGroupSens == i,],
                by(imp_long_excl[imp_long_excl$SFQGroupSens == i,], .imp,
                   function(x) c(mean(x[[variable]]),sd(x[[variable]]))))
    age <- round(Reduce("+",fit)/length(fit),digits = 2)
    age <- paste0(age[1], ' (', age[2], ')')
    numitem <- cbind(numitem, age)
  }
  colnames(numdescr) <- colnames(numitem)
  numdescr <- rbind(numdescr, numitem)
}
numdescr <- numdescr[-1,]
numdescr <- data.frame(numdescr)
rownames(numdescr)<-numlist
colnames(numdescr)<-c('low','mod','high')
```

Categorical variable descriptive for loop.

```
Questcat <- c('MaritalSt2G', 'Children', 'Educ2G',
              'OccupationalStatus','PreviousCancer',
              'Psychiatric', 'PsychDrugs',
              'PriorBreastSurg','Diagnosis','Neoadjuvant',
              'Mastectomy', 'BreastReconstruction', 'BCT', 'SN', 'ALND',
              'Hospital',
              'SporadicPain', 'ChronicPain')

Descriptives_Questionnaires <- matrix(nrow = 1, ncol = 6)
for (i in Questcat){
  imp_long_excl[[i]] <- as.factor(imp_long_excl[[i]])
  Questitems <- c(with(imp_long_excl,
                       by(imp_long_excl, .imp, function(x)
                         c(length((!is.na(x[[i]]))),
                           round(length((x[[i]][x[[i]]==0]))/length(!is.na(x[[i]]))*1
00,2),
                           round(length((x[[i]][x[[i]]==1]))/length(!is.na(x[[i]]))*1
00,2),
                           round(length((x[[i]][x[[i]]==2]))/length(!is.na(x[[i]]))*1
00,2),
                           round(length((x[[i]][x[[i]]==3]))/length(!is.na(x[[i]]))*1
00,2),
                           round(length((x[[i]][x[[i]]==4]))/length(!is.na(x[[i]]))*1
00,2)
                         ))))
  Questitem<- Reduce("+",Questitems)/length(Questitems)
  Descriptives_Questionnaires <- rbind(Descriptives_Questionnaires,
                                       round(Questitem, digits = 2))}
Descriptives_Questionnaires <- data.frame(Descriptives_Questionnaires)
Descriptives_Questionnaires <- Descriptives_Questionnaires[-c(1),]
colnames(Descriptives_Questionnaires) <- c('N', '%0','%1', '%2', '%3', '%4')
rownames(Descriptives_Questionnaires) <- Questcat
```

ANOVA - variable classification

```
for (i in c('Children', 'PreviousCancer', 'Psychiatric', 'PsychDrugs',
            'PriorSurg','Neoadjuvant', 'Hospital', 'Mastectomy',
            'BreastReconstruction','BCT', 'SN', 'ALND', 'ChronicPain',
            'SporadicPain','Age', 'HADSAnxiety',
            'HADSDepression','IEQ', 'LOT_R', 'SocialSupport',
            'PainExpectation')){
  imp_long_excl[[i]] <- as.numeric(imp_long_excl[[i]])
}
```

ANOVA

```
ANOVA_list <- c('Age','PainExpectation','HADSAnxiety','HADSDepression','IEQ','LOT_
R','SocialSupport','PriorSurg')

# Games Howell Post Hoc has been calculated manually due to problems with
# incorporation in for loop
imp_long_excl$SFQGroupSens <- as.factor(imp_long_excl$SFQGroupSens)
anovaOverview<- c()
for (x in ANOVA_list){
  LevT <- c()
  for (i in c(1:2)){
    dataANOVA <- imp_long_excl[imp_long_excl$.imp == i,]
    fit = leveneTest(dataANOVA[[x]] ~ dataANOVA$SFQGroupSens)
    LevT <- rbind(LevT,fit$`Pr(>F)`)}

  # Levene's significant -> welchF + Games Howell + varequal false
  fitLong = data.frame(matrix(ncol=7))
  if (mean(LevT[,1]) < 0.05) {
    for (i in c(1:2)){
      fit <- oneway.test(dataANOVA[[x]] ~ dataANOVA$SFQGroupSens,
                         var.equal = FALSE)
      #PostHoc <- games_howell_test(data = dataANOVA, [[x]] ~ SFQGroup, conf.level =
0.95, detailed = FALSE)

      fitLong <- rbind(fitLong, c(fit$parameter[1], fit$parameter[2],
                                  fit$statistic, fit$p.value, NA,NA,NA
                                  #PostHoc$p.adj[1],PostHoc$p.adj[2],PostHoc$p.adj[3]
      ))}}

  # Levene's not significant -> ANOVA F + Tukey + varequal true
  if (mean(LevT[,1]) > 0.05) {
    for (i in c(1:2)){
      fit <- aov(dataANOVA[[x]] ~ dataANOVA$SFQGroupSens)
      ANOVA <- summary(fit)
      Tukey <- TukeyHSD(fit)
      fitLong <- rbind(fitLong, c(ANOVA[[1]][["Df"]][1], ANOVA[[1]][["Df"]][2],
                                  ANOVA[[1]][["F value"]][1],
                                  ANOVA[[1]][["Pr(>F)"]][1],
                                  Tukey$`dataANOVA$SFQGroupSens`[2,"p adj"],
                                  Tukey$`dataANOVA$SFQGroupSens`[3,"p adj"],
                                  Tukey$`dataANOVA$SFQGroupSens`[1,"p adj"]))}}

  fitLong <- fitLong[-1,]
  colnames(fitLong) <- c('df1', 'df2', 'F', 'p', 'G1', 'G2', 'G3')
  stat <- paste0('F(',mean(fitLong$df1), ',', mean(fitLong$df2), ')',
                 round(mean(fitLong$F),digits = 3))
  p <- round(mean(fitLong$p), digits = 3)
  PH <- cbind(round(mean(fitLong$G1),digits = 3),
              round(mean(fitLong$G2),digits = 3),
              round(mean(fitLong$G3),digits = 3))
  item <- cbind(stat,p,PH)

  anovaOverview <- rbind(anovaOverview, item)
}
anovaOverview2 <- data.frame(anovaOverview)
```

```
PostHoc <- games_howell_test(data = dataANOVA,  HADSDepression ~ SFQGroupSens, conf.l
evel = 0.95, detailed = FALSE)
anovaOverview2[4,c(3:5)] <- round(c(PostHoc$p.adj[2],PostHoc$p.adj[3],PostHoc$p.adj
[1]),3)
PostHoc <-games_howell_test(data = dataANOVA,  IEQ ~ SFQGroupSens, conf.level = 0.95,
detailed = FALSE)
anovaOverview2[5,c(3:5)] <- round(c(PostHoc$p.adj[2],PostHoc$p.adj[3],PostHoc$p.adj
[1]),3)
PostHoc <-games_howell_test(data = dataANOVA,  PriorSurg ~ SFQGroupSens, conf.level =
0.95, detailed = FALSE)
anovaOverview2[8,c(3:5)] <- round(c(PostHoc$p.adj[2],PostHoc$p.adj[3],PostHoc$p.adj
[1]),3)

colnames(anovaOverview2) <- c('stat', 'p', '1vs3', '2vs3', '1vs2')
rownames(anovaOverview2) <- ANOVA_list
```

Chi-Square - variable classification

```
ChiVars <- c('MaritalSt2G', 'Children', 'Educ2G', 'OccupationalStatus','PreviousCance
r', 'Psychiatric', 'PsychDrugs','PriorBreastSurg','Diagnosis','Neoadjuvant','Surger
y','BCT', 'SN','ALND','Hospital','ChronicPain', 'SporadicPain','SFQGroupSens')

for (i in ChiVars){
  imp_long_excl[[i]] <- as.numeric(imp_long_excl[[i]])
}
```

Chi-Square

```
ChiSquares <- data.frame(matrix(ncol = 5))
Chi_var <- c('MaritalSt2G', 'Children', 'Educ2G', 'OccupationalStatus',

             'PreviousCancer', 'Psychiatric', 'PsychDrugs','PriorBreastSurg',

             'Diagnosis','Neoadjuvant','Surgery','BCT', 'SN','ALND','Hospital',

             'ChronicPain', 'SporadicPain')
for (variable in Chi_var){
  chi_total <- c()
  chi_item_mean <- c()
  for (i in c(1:2)){
    chi_DF <- imp_long_excl[imp_long_excl$.imp == i,]
    M <- as.table(rbind(c(length(chi_DF[[variable]][chi_DF$SFQGroupSens == 1 & chi_DF
[[variable]] == 1]),
                          length(chi_DF[[variable]][chi_DF$SFQGroupSens == 2 & chi_DF
[[variable]] == 1]),
                          length(chi_DF[[variable]][chi_DF$SFQGroupSens == 3 & chi_DF
[[variable]] == 1])),
                        c(length(chi_DF[[variable]][chi_DF$SFQGroupSens == 1 & chi_DF
[[variable]] == 2]),
                          length(chi_DF[[variable]][chi_DF$SFQGroupSens == 2 & chi_DF
[[variable]] == 2]),
                          length(chi_DF[[variable]][chi_DF$SFQGroupSens == 3 & chi_DF
[[variable]] == 2]))))
    dimnames(M) <- list(MaritalSt = c("Y", "N"),
                        FearGroup = c("low","moderate", "high"))
    chisq <- chisq.test(M)
    chisqPH <- chisq.posthoc.test(M, method = "bonferroni")

    if (any(chisq$expected<=5) == TRUE){
      Fis_test <- fisher.test(M)
      if (Fis_test$p > 0.05){
        chi_item <- c(chisq$parameter,chisq$statistic,
                      chisq$p.value,chisqPH$mod[4],
                      chisqPH$moderate[4],chisqPH$high[4])
      }
      if (Fis_test$p <= 0.05){
        chi_item <- c(NA,NA,NA,NA,NA,NA,NA)
      }
    }
    if (any(chisq$expected<=5) == FALSE){
      chi_item <- c(chisq$parameter,chisq$statistic,
                    chisq$p.value,chisqPH$mod[4],
                    chisqPH$moderate[4],chisqPH$high[4])
    }
    chi_total <- data.frame(rbind(chi_total, chi_item))
    colnames(chi_total) <- c('df', 'statistic', 'p', 'LowP', 'ModP', 'HighP')
  }
  chi_item_mean <- c(paste0('X2(', round(mean(chi_total$df), digits = 3), ',N=195)=',
                            round(mean(chi_total$statistic),digits = 3)),
                     round(mean(chi_total$p),digits = 3),
                     mean(chi_total$LowP),mean(chi_total$ModP),mean(chi_total$HighP))

  ChiSquares <- rbind(ChiSquares, chi_item_mean)
```

```
}
ChiSquares <- ChiSquares[-1,]
rownames(ChiSquares)<-c(Chi_var)
colnames(ChiSquares)<-c('Statistic', 'p', 'LowP', 'ModP', 'HighP')

# remove all post hoc tests where ChiSquares$p > 0.05
ChiSquares[ChiSquares$p>.05,c(3:5)] <- NA
```

P adjust for multiple comparison

Similar to the main analysis, p-values were adjusted using Benjamini-Hochberg correction.

```
PadjustedValues <- p.adjust(c(anovaOverview2$p,ChiSquares$p), method = "BH")

Padjusted <- data.frame(variable = c(rownames(anovaOverview2),rownames(ChiSquares)),
                        oldP = round(c(as.numeric(anovaOverview2$p),as.numeric(ChiSqu
ares$p)),3),
                        newP = c(round(PadjustedValues,3)))
Padjusted$changedSig[Padjusted$oldP <= .05 & Padjusted$newP > .05] <- 'Changed!'
```

Sensitivity analysis 2

A new datafile was used which includes all participants (included + excluded).

```
dataSensitivity <- read_sav('Drive:/Path/Filename_All_Participants.sav')
```

Grouping variables were calculated

```
dataSensitivity$BCT[dataSensitivity$BCTOnco == 1 | dataSensitivity$BCTOnly == 1] <- 2
dataSensitivity$BCT[is.na(dataSensitivity$BCT)] <- 1
```

Statistical for loop to compare included and excluded participant groups on basic characteristics

```
VariableList <- c('Age','BCvsDCIS', 'Mastectomy', 'BCT', 'SN', 'ALND')
Sensitivity <- data.frame(matrix(ncol = 3))

for (i in VariableList){
  if (i == 'Age'){
    test <- t.test(dataSensitivity[[i]][dataSensitivity$Group == 1],dataSensitivity
[[i]][dataSensitivity$Group == 2])
    G1 <- paste0(round(mean(dataSensitivity[[i]][dataSensitivity$Group == 1], na.rm=
T),2), '(',
                 round(sd(dataSensitivity[[i]][dataSensitivity$Group == 1], na.rm=T),
2), ')')
    G2 <- paste0(round(mean(dataSensitivity[[i]][dataSensitivity$Group == 2], na.rm=
T),2), '(',
                 round(sd(dataSensitivity[[i]][dataSensitivity$Group == 2], na.rm=T),
2), ')')
    stat <- paste0('T(', round(test$parameter,2),')=', round(test$statistic,2), ', p
=', round(test$p.value,3))
  }
  if (i != 'Age'){
    test <- chisq.test(dataSensitivity$Group, dataSensitivity[[i]])

    G1 <- paste0(length(dataSensitivity$Group[dataSensitivity[[i]]==1 & !is.na(dataSe
nsitivity[[i]]) & dataSensitivity$Group == 1]), '(',
           round(length(dataSensitivity$Group[dataSensitivity[[i]]==1 & !is.na(dataSe
nsitivity[[i]])& dataSensitivity$Group == 1])/length(dataSensitivity$Group[!is.na(dat
aSensitivity[[i]])& dataSensitivity$Group == 1])*100,2), '%)')
    G2 <- paste0(length(dataSensitivity$Group[dataSensitivity[[i]]==1 & !is.na(dataSe
nsitivity[[i]])& dataSensitivity$Group == 2]), '(',
           round(length(dataSensitivity$Group[dataSensitivity[[i]]==1 & !is.na(dataSe
nsitivity[[i]])& dataSensitivity$Group == 2])/length(dataSensitivity$Group[!is.na(dat
aSensitivity[[i]])& dataSensitivity$Group == 2])*100,2), '%)')
    stat <- paste0('X2(', round(test$parameter,2),')=', round(test$statistic,2), ', p
=', round(test$p.value,3))
  }
  Sensitivity <- rbind(Sensitivity, c(G1, G2,stat))
}
Sensitivity <- Sensitivity[-1,]
rownames(Sensitivity) <- VariableList
colnames(Sensitivity) <- c('included', 'excluded', 'stat')
```
