# Supplementary material for: A cross-sectional study of fear of surgery in female breast cancer patients: Prevalence, severity, and sources, as well as relevant differences among patients experiencing high, moderate, and low fear of surgery
Source: PLoS One. 2023 Jun 23;18(6):e0287641. doi: 10.1371/journal.pone.0287641 (PMC10289430; doi:10.1371/journal.pone.0287641)
Supplement: S1 Table — (PDF) [file pone.0287641.s003.pdf]

'A cross-sectional study of fear of surgery in female breast cancer patients:
Prevalence, severity, and sources, as well as relevant differences among patients experiencing high, moderate, and low fear of surgery'

Sophia Engel[1], Henrik Børsting Jacobsen[1,2], Silje Endresen Reme[1,2]

[1] The Mind Body Lab, Department of Psychology, University of Oslo, Oslo, Norway
[2] Department of Pain Management and Research, Oslo University Hospital, Oslo, Norway

S1 Table. One-way ANOVA's assessing differences in mean scores of the Surgical Fear Questionnaire (SFQ) between fear groups (high, medium, low).

	**Fear**									**Post-hoc Analyses**		
	**High (≥36)**		**Moderate (15-35)**		**Low (≤14)**		**Test of linear trend**	***p***	**Effect size**			
	N	*M(SD)*	*N*	*M(SD)*	*N*	*M(SD)*	*F*(*df*)		ω^2	High vs. Low	High vs. Moderate	Moderate vs. Low
**Variable**												
SFQ Total	47	47.5(10.08)	98	25.02(6.03)	50	6.89(4.08)	*F*(1,192)=826.48	< .001	0,810	< .001	< .001	< .001

Note. A significant linear trend could be observed. Throughout low to high fear group the mean SFQ score increased significantly. Above this, means of all groups differed significantly from each other (all post-hoc comparisons p <.001). The observed effect was large ($\omega^2 \geq .14$) (1,2).

References

1. Generalized Eta and Omega Squared Statistics: Measures of Effect Size for Some Common Research Designs. - PsycNET [Internet]. [cited 2022 Jun 5]. Available from: https://psycnet.apa.org/doiLanding?doi=10.1037%2F1082-989X.8.4.434

2. An Unbiased Correlation Ratio Measure | PNAS [Internet]. [cited 2022 Jun 5]. Available from: https://www.pnas.org/doi/abs/10.1073/pnas.21.9.554
