# Supplementary material for: A cross-sectional study of fear of surgery in female breast cancer patients: Prevalence, severity, and sources, as well as relevant differences among patients experiencing high, moderate, and low fear of surgery
Source: PLoS One. 2023 Jun 23;18(6):e0287641. doi: 10.1371/journal.pone.0287641 (PMC10289430; doi:10.1371/journal.pone.0287641)
Supplement: S2 Table — (PDF) [file pone.0287641.s004.pdf]

‘**A cross-sectional study of fear of surgery in female breast cancer patients**: Prevalence, severity, and sources, as well as relevant differences among patients experiencing high, moderate, and low fear of surgery’

Sophia Engel[1], Henrik Børsting Jacobsen[1,2], Silje Endresen Reme[1,2]

[1] The Mind Body Lab, Department of Psychology, University of Oslo, Oslo, Norway
[2] Department of Pain Management and Research, Oslo University Hospital, Oslo, Norway

S2 Table. Sensitivity analyses: Comparison of eligible patients - participating versus non-participating.

	**Patients**			
	**Participating**	**Not participating**	**Statistical test**	***p***
	M(SD)/N(%)	M(SD)/N(%)	t(*df*)/ χ^2(*df, N*)	
**Variable**				
Age	53.59(9.73)	55.52(10.11)	*t*(316)= -1.692	.092
Invasive BC	173(89.2%)	96(87.3%)	χ^2 (1,304)=0.098	.755
Non-invasive BC	21(10.8%)	14(12.7%)		
BCT	132(68.0%	84(67.2%)	χ^2 (1,307)=0.421	.517
Mastectomy	58(29.9%)	36(31.9%)	χ^2 (1,307)=3.167	.160
SN	160(82.5%)	91(80.5%)	χ^2 (1,307)=0.074	.786
ALND	29(14.9%)	19(17.0%)	χ^2(1,306)=0.092	.761

Note. Displayed are the results of independent samples t-tests and chi-square tests comparing eligible patients who participated in the present study to those who didn’t.

Abbreviations
BC, breast cancer; SN, sentinel node procedure; ALND, axillary lymph node dissection
