# Supplementary material for: A cross-sectional study of fear of surgery in female breast cancer patients: Prevalence, severity, and sources, as well as relevant differences among patients experiencing high, moderate, and low fear of surgery
Source: PLoS One. 2023 Jun 23;18(6):e0287641. doi: 10.1371/journal.pone.0287641 (PMC10289430; doi:10.1371/journal.pone.0287641)
Supplement: S3 Table — (PDF) [file pone.0287641.s005.pdf]

‘**A cross-sectional study of fear of surgery in female breast cancer patients**:
Prevalence, severity, and sources, as well as relevant differences among patients experiencing high, moderate, and low fear of surgery’

Sophia Engel[1], Henrik Børsting Jacobsen[1,2], Silje Endresen Reme[1,2]
[1] The Mind Body Lab, Department of Psychology, University of Oslo, Oslo, Norway
[2] Department of Pain Management and Research, Oslo University Hospital, Oslo, Norway

S3 Table. Sensitivity analyses: Comparison of two different cutoff scores to indicate high fear of surgery.

	**Fear**			**ANOVA/Chi-square**		**Post-hoc Analyses**		
	**High (≥36) N=50**	**Moderate (15-35) N=98**	**Low (≤14) N=47**					
	**High (≥44) N=29**	**Moderate (15-43) N=119**	**Low (≤14) N=47**					
	M(SD)/N(%)	M(SD)/N(%)	M(SD)/N(%)	*F(df, df)*/χ^2*(df, N)*	p	High vs. Low	High vs. Moderate	Moderate vs. Low
**Sociodemographic variables**								
Age	52.63(9.61)	52.45(10.10)	56.96(8.46)	$F(2,192)=3.87$	0.083	.097	.961	.024
	53.81(10.02)	52.19(9.89)	56.96(8.46)	$F(2,192)=4.23$	0.067	.464	.547	.012
Marital status (partner or married)	31(62.00%)	70(71.43%)	31(65.96%)	$\chi^2(2,N=195)=1.43$	0.718			
	19(65.92%)	82(68.91%)		$\chi^2(2,N=195)=0.20$	0.914			
Children	42(84.00%)	82(83.49%)	38(80.85%)	$\chi^2(2,N=195)=0.21$	0.937			
	25(86.21%)	99(83.01%)		$\chi^2(2,N=195)=0.36$	0.914			
Education (higher or university)	37(74.00%)	72(73.66%)	38(80.85%)	$\chi^2(2,N=195)=0.98$	0.764			
	22(75.86%)	87(73.22%)		$\chi^2(2,N=195)=1.09$	0.804			
Working	11(21.02%)	21(21.43%)	9(19.15%)	$\chi^2(2,N=195)=0.13$	0.937			
	23(80.31%)	93(78.15%)		$\chi^2(2,N=195)=0.26$	0.914			
**Medical history**								
Cancer	4(8.00%)	16(16.33%)	5(10.64%)	$\chi^2(2,N=195)=2.32$	0.595			
	2(6.90%)	18(15.13%)		$\chi^2(2,N=195)=1.68$	0.765			
Psychiatric diagnoses	4(7.22%)	5(5.10%)	1(2.13%)	$\chi^2(2,N=195)=1.41$	0.718			
	2(5.55%)	7(5.88%)		$\chi^2(2,N=195)=1.19$	0.804			
Use of antidepressants, antipsychotics and/or benzodiazepines	8(16.00%)	15(15.31%)	6(12.77%)	$\chi^2(2,N=195)=0.23$	0.937			
	4(13.79%)	19(15.97%)		$\chi^2(2,N=195)=0.30$	0.914			

Sophia Engel[1], Henrik Børsting Jacobsen[1,2], Silje Endresen Reme[1,2]
[1] The Mind Body Lab, Department of Psychology, University of Oslo, Oslo, Norway
[2] Department of Pain Management and Research, Oslo University Hospital, Oslo, Norway

Number of surgeries	2.18(2.34)	1.83(1.58)	2.91(2.52)	$F_{Welch}(2,84.60)=0.07$	0.083	.301	.602	.023
	1.76(1.94)	1.99(1.86)	2.91(5.52)	$F_{Welch}(2,63.50)=3.080$	0.189	.070	.829	.066
Breast surgery	2(4.00%)	24(24.49%)	8(17.02%)	$\chi^2(2,N=195)=9.66$	0.033		≤.05	
	1(3.45%)	25(21.01%)		$\chi^2(2,N=195)=5.00$	0.256			
**Current diagnosis and treatment**								
Primary non-invasive BC	7(14.00%)	8(8.16%)	6(12.77%)	$\chi^2(2,N=195)=1.21$	0.718			
	4(13.79%)	11(9.24%)		$\chi^2(2,N=195)=0.67$	0.914			
Primary invasive BC	42(83.76%)	84(85.71%)	40(85.11%)					
	24(82.3%)	103(85.71%)						
Primary BC + Metastases	(0.00%)	0(0.00%)	1(2.13%)					
	(0.00%)	0(0.00%)						
Recurrent BC	1(2.00%)	4(4.08%)	0(0.00%)					
	1(3.45%)	4(3.36%)						
Recurrent BC + Metastases	0 (0.00%)	2(2.04%)	0(0.00%)					
	0 (0.00%)	2(1.68%)						
Neoadjuvant treatment	17(33.22%)	32(32.70%)	17(36.17%)	$\chi^2(2,195)=0.18$	0.937			
	10(33.83%)	39(33.77%)		$\chi^2(2,N=195)=0.18$	0.914			
Mastectomy with/without primary reconstruction	18(36.0%)	29(29.60%)	11(23.40%)	$\chi^2(2,195)=2.31$	0.595			
	11(37.00%)	37(32.09%)		$\chi^2(2,N=195)=1.61$	0.765			
Breast-conserving surgery with/without oncoplasty	31(62.00%)	65(66.30%)	36(76.60%)	$\chi^2(2,195)=2.20$	0.595			
	19(64.62%)	78(65.55%)		$\chi^2(2,N=195)=2.01$	0.763			
SN	38(76.98%)	83(84.69%)	39(82.98%)	$\chi^2(2,195)=1.26$	0.718			
	22(74.69%)	100(84.03%)		$\chi^2(2,N=195)=1.63$	0.765			
ALND	11(21.12%)	14(14.29%)	5(10.64%)	$\chi^2(2,195)=2.24$	0.595			
	7(22.76%)	18(15.13%)		$\chi^2(2,N=195)=2.53$	0.707			
Hospital	37(73.26%)	60(61.22%)	25(53.19%)	$\chi^2(8,N=195)=4.38$	0.318			
	20(68.03%)	77(64.71%)		$\chi^2(2,N=195)=2.29$	0.730			

Sophia Engel[1], Henrik Børsting Jacobsen[1,2], Silje Endresen Reme[1,2]
[1] The Mind Body Lab, Department of Psychology, University of Oslo, Oslo, Norway
[2] Department of Pain Management and Research, Oslo University Hospital, Oslo, Norway

**Pain**								
Sporadic	5(10.00%)	14(14.29%)	4(8.51%)	$\chi^2(2,N=195)=1.49$	0.718			
	3(10.34%)	17(13.92%)		$\chi^2(2,N=195)=1.16$	0.804			
Chronic	21(42.00%)	35(35.71%)	20(42.55%)	$\chi^2(2,N=195)=0.88$	0.765			
	10(34.48%)	46(38.66%)		$\chi^2(2,N=195)=0.50$	0.914			
Expected postsurgical pain	6.11(1.85)	4.73(1.81)	3.87(1.9)	$F(2,192)=18.608$	<.001	<.001	<.001	.026
	6.44(1.83)	4.90(1.83)	3.87(1.9)	$F(2,192)=17.432$	<.001	<.001	<.001	.005
**Psychological measures**								
HADS-A	9.18(3.72)	7.19(3.83)	3.69(3.25)	$F(2,192)=27.89$	<.001	<.001	.006	<.001
	9.55(3.13)	7.45(3.96)	3.69(3.25)	$F(2,192)=26.485$	<.001	<.001	.018	<.001
HADS-D	4.65(2.94)	3.61(2.76)	1.75(1.96)	$F_{Welch}(2,107.01)=19.63$	<.001	<.001	.102	<.001
	5.28(2.33)	3.64(2.89)	1.74(1.96)	$F_{Welch}(2,75.04)=26.036$	<.001	<.001	.006	<.001
IEQ	8.34(4.19)	6.44(3.99)	2.66(2.78)	$F_{Welch}(2,107.55)=38.06$	<.001	<.001	.025	<.001
	8.34(3.73)	6.78(4.19)	2.66(2.78)	$F_{Welch}(2,72.58)=37.892$	<.001	<.001	.127	<.001
LOT-R	15.45(3.53)	17.08(3.94)	19.02(3.85)	$F(2,192)=10.59$	<.001	<.001	.030	.016
	15.02(3.54)	16.90(3.87)	19.02(3.85)	$F(2,192)=10.507$	<.001	<.001	.037	.005
Perceived Social Support	25.72(5.77)	25.68(5.86)	27.44(5.68)	$F(2,192)=1.77$	.433	.291	.997	.173
	25.36(5.60)	25.75(5.86)	27.49(5.68)	$F(2,192)=1.829$	.453	.256	.934	.191

Note. This table presents the comparisons between groups. Results based on reported cutoffs (≤14= little, 15-35= moderate and ≥ 36= high fear) are written in black. In red, results based on a higher cutoff for high fear (≤14= little, 15-43= moderate and ≥ 44= high fear). Despite the differences in cutoffs, significances are largely similar.

Abbreviations
vs, versus; HADS-A/D, Hospital Anxiety and Depression Scale Anxiety/Depression; IEQ, Injustice Experience Questionnaire; LOT-R, Life Orientation Test-Revised; BC, breast cancer; SN, sentinel node procedure; ALND axillary lymph node dissection
