# Supplementary figures and images for: A cross-sectional study of fear of surgery in female breast cancer patients: Prevalence, severity, and sources, as well as relevant differences among patients experiencing high, moderate, and low fear of surgery
Source: PLoS One. 2023 Jun 23;18(6):e0287641. doi: 10.1371/journal.pone.0287641 (PMC10289430; doi:10.1371/journal.pone.0287641)

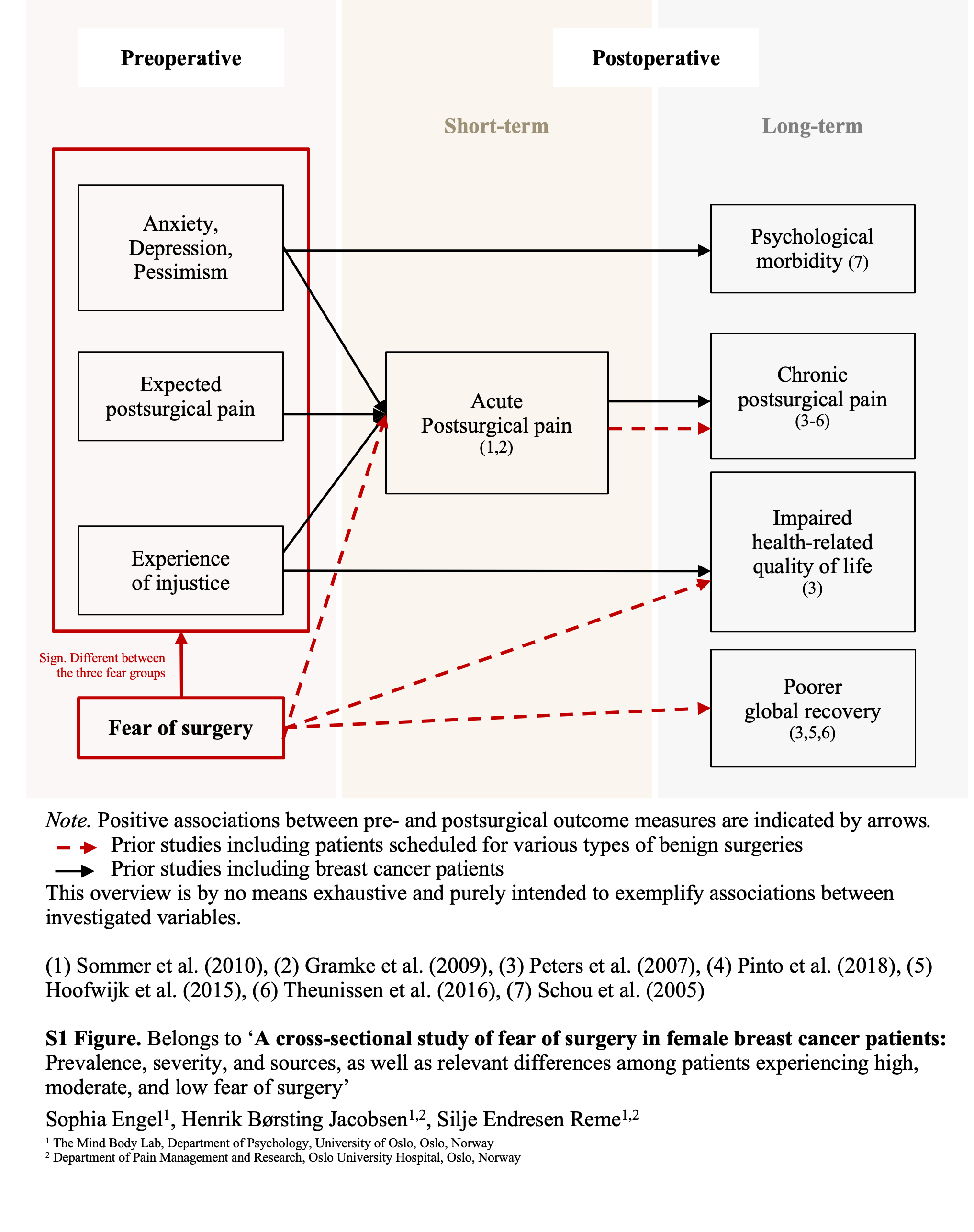

Supplement: S1 Fig — (TIF) [file pone.0287641.s006.tif]
