# Supplementary material for: A cross-sectional study of fear of surgery in female breast cancer patients: Prevalence, severity, and sources, as well as relevant differences among patients experiencing high, moderate, and low fear of surgery
Source: PLoS One. 2023 Jun 23;18(6):e0287641. doi: 10.1371/journal.pone.0287641 (PMC10289430; doi:10.1371/journal.pone.0287641)
Supplement: Attachment — Submitted filename: S.Engel_Response to Reviewers.pdf [file pone.0287641.s007.pdf]

UiO : Department of Psychology
University of Oslo

Alejandro Dominguez-Rodriguez, PhD
Academic Editor
PLOS ONE

Date: 26 March 2023

Dear Editor-in-Chief

Thank you so much for considering and reviewing our research report, '***A cross-sectional study of fear of surgery in female breast cancer patients***: *Prevalence, severity, and sources, as well as relevant differences among patients experiencing high, moderate, and low fear of surgery*', which is as far as we know the first study to systematically investigate fear of surgery among female breast cancer patients.

We are very grateful for the constructive feedback we received. We carefully considered and answered to each reviewer's comments and adapted our manuscript accordingly (using tracked changes). We genuinely believe that the suggestions improved the quality and clarity of our article.

Already in advance, thank you for your time and for considering our research report. We hope that our revised manuscript will meet the high standards of *PLOS ONE* and are very much looking forward to your response.

Sincerely yours,

Sophia Engel (corresponding author)

On behalf of all the authors

Postal address: Postboks 1094 Blindern, 0317 Oslo
E-mail: ekspedisjonen@psykologi.uio.no
www.sv.uio.no/psi/english/

Line numbers & abbreviations

Line numbers: All line numbers refer to lines in the manuscript including 'tracked changes'.
Abbreviations: SFQ; Surgical Fear Questionnaire

Data availability

Dear all,
As indicated in the data availability statement in the submission history, it is unfortunately not possible to make the data underlying our manuscript fully available yet. As our study uses privacy sensitive patient data, data need to be anonymized before they can be accessed. Since the cross-sectional data analyzed in the present study stem from a larger longitudinal trial, the PREVENT trial, data will only be anonymized and made accessible after the trial is completed. This is also described in the PREVENT trial's protocol which was published in PLOS One as well [1]. However, to increase the transparency, we published the 'R' script underlying the analyses of the present study. Moreover, as the PREVENT trial is about to be completed (end of March 2023) the anonymized data will be made available in the close future and can be accessed as described in the data availability statement.

Quality of figures – PACE diagnostic tool

All figures meet the requirements stated in the PLOS One submission guidelines. The by PLOS One suggested Preflight Analysis and Conversion Engine (PACE) digital diagnostic tool was used to confirm appropriate quality.

Reviewer #1 – comments & answers

Dear Author,
Thank you for sharing your interesting study. Regarding the manuscript that you submitted there are some points that should be considered:

Comment from reviewer	Answer from author
1- Abstract:<br>• Please focus the abstract on your study and your results<br>• Provide in the abstract an informative and balanced summary of what was done and what was found.	Thanks a lot for your feedback on our abstract. We do think that the abstract we wrote closely focuses on our study, i.e., the cross-sectional study we conducted to investigate fear of surgery among female breast cancer patients.<br><br>It might have been confusing that we reported scores for anxiety, depression, experience of injustice, and dispositional optimism questionnaires without mentioning the individual questionnaires in the methods section of the abstract. Given the word limit of 300 words we initially summarized the different psychological outcomes with the wording 'psychological questionnaires'. To increase the clarity of our abstract we rewrote the methods section. We hope that this makes it easier to understand that the abstract is focused on the present study only and that the adaptations also add to our abstract being an informative and balanced summary of what has been done and found.
2- Introduction:<br>• Explain the scientific background and rationale for the investigation being reported	In response to your feedback, as well as to that of another reviewer, we adapted the introduction in a way that hopefully increased its clarity and facilitates a better understanding.<br><br>The introduction clearly follows a deductive structure. It starts broadly by describing the aversive effects of a breast cancer diagnosis and preoperative anxiety among breast cancer patients. It then goes into more detail describing that fear of surgery is one aspect of preoperative anxiety and that it is experienced by, but has not thoroughly been investigated in, breast cancer patients. Finally, we end by stating the aversive consequences that have been associated with fear of surgery, underlining the importance of investigating this topic in greater detail in breast cancer patients. We hope that due to the adaptations we made, the gaps in knowledge our study fills are more easily apparent now.

3- Methods:<br>• Explain how the study size was arrived<br>• Present key elements of study design early in the paper<br>• Describe any efforts to address potential sources of bias	**Sample size:**<br>In our manuscript we pick up upon this point in the limitations section (lines 445-447). We give arguments for why an a priori calculation of the sample size and thus power of our study was not possible, as well as for why we refrained from reporting post-hoc power. Below a short summary of the arguments:<br>• **A priori sample size/power calculation:** As the present study is using cross-sectional data from a longitudinal trial the sample size was calculated with respect to the primary outcome of that larger trial (chronic post-surgical pain, measured by means of the 'Numeric Rating Scale' (NRS); for further detail see [1]).<br>• **Post-hoc power calculation**: Not reported as this only would have mirrored already presented p-values [2–4].<br>In summary, given the large group sizes used in the present study we argue our study was sufficiently powered.<br><br>**Key elements of the study design early in the paper:**<br>Key information on the study design is first presented in the last paragraph of the introduction (line 96, 'cross-sectionally'). More detailed information on the study design is then given in the first three paragraphs of the methods section ('Setting and participants'). We adapted the wording in the first paragraph of the methods section to indicate more clearly that the present study uses cross-sectional data that stem from a larger trial, the PREVENT trial (see lines: 111, 119, 120). We hope that these adaptations facilitate an improved understanding of the study design we used and believe that key information on the study design is presented as early as possible.<br><br>**Bias:** Below information on aspects of bias already addressed in our manuscript, as well as aspects we added after receiving your feedback. Thanks a lot!<br>• **Selection bias:** Lines 433-436 - We describe that the fact that participation in the PREVENT trial necessitated an interest in hypnosis, iACT and mindfulness might have introduced selection bias. We admit, that even though participants and eligible non-participants did not differ in their age or cancer-related variables, psychological differences cannot be excluded.<br>• **Sampling bias:**<br>Lines 431-433 - We describe that because decreasing work-disability was a major goal of the PREVENT trial, patients older than 70 years were excluded and admit that this limits the generalizability of our results to such patients.<br>Lines 436-439 - Moreover, we emphasize that conclusions of the present work are confined to female breast cancer patients of Northern European descent. We argue, that because health-care systems, cultural stigma and knowledge about breast cancer surgery differ greatly on a global scale, it is likely that fear of surgery will do so as well.<br>• **Interviewer and response bias:** Interviewer bias was prevented by having patients answer the questionnaires online from at home.<br>• **Publication bias:** Publication bias was prevented by publishing the protocol of the PREVENT trial before publishing results [1]. The present study is mentioned as objective number 9 among the secondary objectives.<br><br>We hope that we could convince you that we addressed bias in a thorough manner in the present study and that this is also reflected in the manuscript.
4- In this study, you have used several questionnaires and items, How have you avoided the fatigue and overlaps of answering all the items at once for these participates in this certain	Thank you for addressing two very important points!<br><br>As described in our manuscript, the present study constitutes part of a larger trial (the PREVENT trial). Measures taken to ***reduce responder burden*** within the frame of the trial were the following:<br>• The questionnaire selection was reviewed by our patient representative and adapted according to their feedback [1]. Furthermore, our user representative estimated that it would take 15-20 minutes to complete all questionnaires and regarded that as acceptable. We tried to specify this point more clearly in the revised manuscript (lines 128-131).

situations?<br>Thank you	• Further, a shorter version of the 'Injustice Experience Questionnaire' (IEQ) containing only 5 of originally 12 items was constructed and validated [1] (lines 182-185).<br>• Where valid, even single items were used to assess the outcome of interest (like for example for 'expected postoperative pain' (lines 165-168).<br><br>Measures taken to prevent ***overlap between questions*** of different questionnaires within the frame of the trial were the following:<br>• All psychological outcomes measured in the PREVENT trial were addressed with one questionnaire only. All included questionnaires have been validated, thus shown to possess sufficient discriminant validity, priorly.<br>• Involvement of our patient representative in the choice of questionnaires assured the relevance of selected questions and questionnaires to the patient population at hand, as well as to prevent unnecessary burdening of participants.

Reviewer #2 – comments & answers

Comment from reviewer	Answer from author
1.This article is the result of a larger study consisting of a Trial. I think it would be more innovative if these results are presented as part of what was obtained in the trial.	We understand that it could be relevant and interesting to present the results of our study in the frame of the larger trial. However, given the large scope of the PREVENT trial, which is the larger trial that the cross-sectional data of the present study stem from, it appeared necessary to report the current study in a separate paper. Given the lack of studies investigating fear of surgery, both in general and in breast cancer patients specifically, the current study is justified as an independent contribution to the field. Further, the research questions we report in the current study were also preregistered in the protocol paper published at PLOS One (objective number 9), with the intention of reporting it as a separate study [1]. The present study has thus been specifically designed to investigate 'fear of surgery' as a potentially modifiable risk factor among female breast cancer patients. We believe that an inclusion of broader results of the trial would distract from the novel and relevant findings we found with respect to fear of surgery among female breast cancer patients in this cross-sectional study.
2. Despite the fact that they made very clear and adequate statistics, the subject has been large studied, thus, lacks novelty. Even in the introduction they talk about other studies that have already studied the subject.	We are afraid that we must disagree on this point. In the introduction we indeed mention prior studies that reported on fear of surgery among female breast cancer patients. Prior to our study, however, there has not been conducted a single study that was specifically focused on investigating fear of surgery among female breast cancer patients in detail. Arguments that support the novelty and relevance of our study are the following:<br><br>Firstly, it is important not to mistake fear of surgery for preoperative anxiety. Whereas the prior is a very specific fear targeted at the surgery and associated processes, preoperative anxiety describes more general state of unspecific anxiety that patients awaiting a surgery might feel. In the introduction, we highlight, that fear of surgery can contribute to such preoperative anxiety, as well as that preoperative anxiety among BC patients has been shown to be associated with a range of adverse peri- and postoperative health-related consequences. A detailed investigation of fear of surgery among female breast cancer patients therefore appeared relevant.<br><br>Secondly, we mention prior studies reporting on fear of surgery among breast cancer patients to highlight their limitations and to emphasize how our study overcomes these. First and foremost, fear of surgery has not been studied as a topic on its own among female BC patients. Prior studies were either qualitative in nature and focused on the preoperative phase in general [5,6] or used only a single item to assess fear of surgery [7–9]. Our study builds on and exceeds these prior studies by being the first to dedicatedly focus on fear of surgery as a primary outcome, as well as to investigate the topic by means of a validated questionnaire. This study set-up enabled us to study fear of surgery among female BC patients in much greater detail (e.g., quantification of fear of individual aspects of surgery and characterization of patients experiencing different levels of such fear) compared to what has been done earlier (fear yes/no).

	Moreover, we advance prior broadly formulated findings by adding more detail to them. One prior study among BC patients for example stated that fear of surgery was associated with poorer mental health before surgery [7]. 'Poorer' mental health is a broad term and was not further specified in that study. Our study advances this finding by reporting on outcomes as anxiety, depression, dispositional optimism, and experience of injustice and how patients experiencing different levels of fear of surgery differ in their experience of these. We thus provide a much more detailed view on patients' mental health in the preoperative phase.<br><br>Further, fear of surgery is malleable. Given great differences in psychological wellbeing among patients experiencing different levels of fear of surgery, we highlight a potential way to classify patients in need of further psychological support in the preoperative phase. The insights gained by the present study thus possess clinical relevance.<br><br>Finally, we acknowledge that there is one study that has employed the SFQ among female breast cancer patients. The SFQ, however, was not a primary outcome in that study. Further, to the best of our knowledge, this study has not been published in a peer-reviewed journal. Until today the results are only described in a PhD dissertation [10].<br><br>We adapted the introduction in a way that will hopefully highlight the above-mentioned gaps more clearly and hope that we can convince you of the novelty and importance of the study we conducted.
3. The main instrument used was not translated and they do not present a validation procedure or reliability data. The fact that it is not validated also means that there are no cut-off points established in the Norwegian population, despite the procedure they carried out to obtain them. In addition, I suggest to specify if it has been used in cancer patients, given the particularities of breast surgery.	Thank you for this comment! The translation of the Surgical Fear Questionnaire (SFQ) is indeed an important point to raise. Based on your comment we took the initial validation analyses we had already conducted (construct validity, internal consistency) further and completed a full validation procedure. We summarized the results of the analyses in a document that we will publish as supplementary information (S1 file). Moreover, we adapted the limitations section accordingly. We now mention the fact that the instrument has been officially validated as an additional strength of the study and removed this point from the limitations section.<br><br>Concerning the *cut-offs for the SFQ* - To the best of our knowledge the present study is the first to describe cut-offs for the SFQ. We feel that we report in sufficient detail how the cut-offs for the fear groups were derived and why we chose to specify them in the way we did. Please let us know if you disagree. Moreover, we believe that cut-offs, such as the ones we chose, will enlarge the clinical applicability of the SFQ in the breast cancer patient population. We are, however, also aware of the limitations associated with the cut-offs we chose and clearly state these as well (lines 212-222 and lines 449-450, 456-457). Moreover, we encourage longitudinal studies on the topic to confirm or reconsider the cut-offs we present in a way that increases their prognostic value.<br><br>Finally, concerning your remark that we should specify whether the instrument has been used among breast cancer patients before, given the particularities of breast surgery – We do specify that the SFQ has been used in one other sample of breast cancer patients and state the very apparent and important limitations of that study (lines 79-83). In addition, the questions of the SFQ have a very general character and the scale has been developed using data from five samples including elective surgery and inpatients undergoing various types of surgery [11]. These arguments underline a suitability of the questionnaire or patients undergoing various types of surgery.
4. I consider it important in the Outcome measures section to explicitly divide with primary outcomes and secondary outcomes	Great idea! We added section headings and hope that this will result in an improved structure of the methods section.

sections.	
5. The instrument to assess pain is specific for fibromyalgia. I consider that it is a limitation since it evaluates pain in parts of the body that is typical of fibromyalgia, leaving aside places in the body that patients with breast cancer may have pain.	Another important point. Indeed, the original 2016 Fibromyalgia (FM) Diagnostic Criteria were developed to diagnose FM. As mentioned in the methods section, however, we used a ***modified*** 10-item version of these criteria that was developed to assess widespread pain. Moreover, we detail the exact content of these 10 items in lines 160-162 - 'Five items assessed whether participants experienced pain in any of the four body quadrants or their neck or back. The other five asked whether such pain had persisted for more than three months.'<br><br>In summary, the instrument asks for pain in any of the four body quadrants + the neck and back as a 5th region and whether such pain is chronic (i.e., has persisted for ≥ 3 months) or not. The questions thus cover the whole body and not just regions specific to fibromyalgia. Accordingly, also those body regions breast cancer patients most often have pain at are covered by the instrument. We would also like to remind that these questions are meant as an assessment of preoperative chronic pain conditions and that (chronic) pain due to breast cancer in the preoperative phase is very rare.<br><br>We agree, the name of the instrument might be misleading, but nevertheless remain convinced that the questionnaire adequately addressed the outcomes we sought to investigate, namely sporadic and chronic pain in any body region.
6.It is not clear why the secondary outcomes were included: anxiety and depression, dispositional optimism, injustice experience and social support. Since in the introduction it is not specified.	Thank you for raising this important question. We specified the choice of secondary outcomes in lines 100-103 in the introduction ('targeted secondary outcomes were chosen based on their *theoretical or priorly established potential* to affect fear of surgery'). In lines 103-106 we then state hypotheses for chosen secondary outcomes, as well as the references to the studies we base our hypotheses on, which implies that secondary outcomes were selected purposefully. We adapted this section, as well as the introduction, to highlight limitations of prior studies more clearly and to show how our study overcomes these, thereby justifying our choice of secondary outcomes. Finally, our patient representative reviewed the selection of secondary outcomes and regarded all of them as relevant for the purpose of the study and the patient population at hand (also specified more clearly in the revised document: lines 128-129).<br><br>In preparation for this study, we also created a table stating the exact reasons for why we included which variable (see Appendix A at the end of the present document). The information it contains, however, was too extensive to state in the article. We also believe that the revised manuscript contains sufficient information on why we chose the outcomes we report (adapted introduction). Nevertheless, we could attach the table as a supplement if that would be regarded as helpful.<br><br>**Detailed argumentation:**<br>In the introduction we cite findings showing that fear of surgery among female breast cancer patients is higher among those with poorer preoperative mental health (lines 64-68). Based on this finding we decided that it would be interesting to further detail 'poorer preoperative mental health' and consequently included questionnaires that are specific for various aspects of mental health (i.e., anxiety, depression, dispositional optimism, experience of injustice). To prevent an overburdening of patients though, we had to limit us and chose to investigate those aspects of mental health we and our patient representative regarded as most important.<br><br>Moreover, based on prior studies that showed that higher fear of surgery was associated with expecting higher postoperative pain, greater acute postoperative pain, as well as a greater likelihood to develop chronic postoperative pain, we decided that it would be interesting to investigate whether fear of surgery would also be associated with preoperative sporadic and/or chronic pain. Accordingly, we included a questionnaire that enabled us to assess sporadic and chronic pain, as well as a single item asking about expected postsurgical pain. Finally, we added a measure of social support as prior studies highlighted that good social support might reduce preoperative anxiety, part of which fear of surgery can be (see lines 403-409).

7. It is excessive to present a figure with the results of each item of the SFQ. I suggest just describing it.	Thank you for this feedback. We understand your concern and removed the figure from the manuscript. To recover the information of the figure, we added two sentences describing its content to the results section (lines 278-284).

Reviewer #3 – comments & answers

This is an interesting research report on the prevalence, severity, and sources of fear of surgery in breast cancer patients in Norway. The study is cross-sectional and includes 204 participants. I have some concerns, which I raise below. I hope the authors consider them as constructive and help them to improve their paper.

Comment from reviewer	Answer from author
Abstract<br>Comment 1. Please indicate in brackets the country of the Hospital (especially useful for non-European readers).	Great remark! We added the country of the hospital (see front page).
Abstract<br>Comment 2. I suggest including percentile 50 in the SFQ in the results section of the abstract.	We agree that the median of the SFQ is a relevant figure to include in the results section of the abstract and adapted the section accordingly – see line 33.
Introduction<br>Comment 3. The authors state that the study is large-scale and the sample is representative of the BC population. However, the sample comes from a single-centre RCT and I have doubts about describing 200 participants as a large sample.	Thank you for this critical remark on the wording we used to describe our study and sample in the introduction of our manuscript.<br><br>Concerning *'largescale'* – The sample includes about 5% of Norway's annual breast cancer population and we think that this can be considered a reasonably large sample. Moreover, previous studies employing the SFQ used samples of similar or even smaller size (n=209 [12], n=150 [13], n=98 [14]), further supporting the adequacy of the size of our sample. Nevertheless, we agree that 'largescale' might have been an overstatement and deleted the word from the abstract and introduction (lines 22 and 94).<br><br>Concerning *'representative'* – We compared data from our sample to the most recent breast cancer statistics from Norway (from 2021; [15]) and concluded that, for the age range of patients we covered, our sample is representative with respect to breast cancer diagnoses and treatment (line 424-425). Moreover, in lines 431-433 we also acknowledge that generalizations drawn from our research do not generalize to breast cancer patients older than 70 years of age, as these were excluded from the study, thereby clearly demarcating the 'representativeness' of our sample.
Methods<br>Comment 4. Line 208. Maybe there is an increased risk of type II error, but also of Type I. I suggest correcting for multiple testing since 23 between-group comparison analysis (ANOVA/chi square) are	Indeed, type I errors are also important to be prevented. In response to your comment, we applied the Benjamini-Hochberg correction in R (to also have the correction included in the 'R' script that we publish with the manuscript) and adapted the p-values accordingly (see also line 232-233). Your assumption was also correct – the main significant differences remained.

conducted. I suggest using the Benjamini-Hochberg correction (https://tools.carbocation.com/FDR). I think that the main significant differences will remain.	
Results<br>Comment 5. Line 242. Please provide the median in text.	We agree that the median is an important statistic to mention in the results section of our manuscript and adapted the text according to your suggestion.

References

[1] Reme SE, Munk A, Holter MTS, Falk RS, Jacobsen HB. Pre- and post-operative psychological interventions to prevent pain and fatigue after breast cancer surgery (PREVENT): Protocol for a randomized controlled trial. PLOS ONE 2022;17:e0268606. https://doi.org/10.1371/journal.pone.0268606.

[2] Hoenig JM, Heisey DM. The Abuse of Power: The Pervasive Fallacy of Power Calculations for Data Analysis. The American Statistician 2001;55:19–24. https://doi.org/10.1198/000313001300339897.

[3] Lakens D. The 20% Statistician: Observed power, and what to do if your editor asks for post-hoc power analyses. The 20% Statistician 2014. http://daniellakens.blogspot.com/2014/12/observed-power-and-what-to-do-if-your.html (accessed June 22, 2022).

[4] Yuan K-H, Maxwell S. On the Post Hoc Power in Testing Mean Differences. Journal of Educational and Behavioral Statistics 2005;30:141–67. https://doi.org/10.3102/10769986030002141.

[5] Drageset S, Lindstrøm TC, Underlid K. Coping with breast cancer: between diagnosis and surgery. Journal of Advanced Nursing 2010;66:149–58. https://doi.org/10.1111/j.1365-2648.2009.05210.x.

[6] Mehnert A, Koch U. Prevalence of acute and post-traumatic stress disorder and comorbid mental disorders in breast cancer patients during primary cancer care: a prospective study. Psycho-Oncology 2007;16:181–8. https://doi.org/10.1002/pon.1057.

[7] Singer S, Blettner M, Kreienberg R, Janni W, Wöckel A, Kühn T, et al. Breast Cancer Patients' Fear of Treatment: Results from the Multicenter Longitudinal Study BRENDA II. Breast Care (Basel) 2015;10:95–100. https://doi.org/10.1159/000381933.

[8] Bruce J, Thornton AJ, Scott NW, Marfizo S, Powell R, Johnston M, et al. Chronic preoperative pain and psychological robustness predict acute postoperative pain outcomes after surgery for breast cancer. Br J Cancer 2012;107:937–46. https://doi.org/10.1038/bjc.2012.341.

[9] Bruce J, Thornton AJ, Powell R, Johnston M, Wells M, Heys SD, et al. Psychological, surgical, and sociodemographic predictors of pain outcomes after breast cancer surgery: A population-based cohort study. PAIN® 2014;155:232–43. https://doi.org/10.1016/j.pain.2013.09.028.

[10] Wittmann V. THE HEALING EFFECT OF PSYCHOSOCIAL INTERVENTIONS IN BREAST CANCER - FROM SURGICAL FEAR TO ENHANCED LONG-TERM QUALITY OF LIFE. Doctoral Thesis. University of Szeged, n.d.

[11] Theunissen M, Peters ML, Schouten EGW, Fiddelers AAA, Willemsen MGA, Pinto PR, et al. Validation of the Surgical Fear Questionnaire in Adult Patients Waiting for Elective Surgery. PLOS ONE 2014;9:e100225. https://doi.org/10.1371/journal.pone.0100225.

[12] Lai Y-H, Huang Y-C, Huang L-T, Chen R-M, Chen C. Cervical Noninvasive Vagus Nerve Stimulation for Migraine and Cluster Headache: A Systematic Review and Meta-Analysis. Neuromodulation: Technology at the Neural Interface 2020;23:721–31. https://doi.org/10.1111/ner.13122.

[13] Kapikiran G, Demir B, Bülbüloğlu S, Saritaş S. THE EFFECT OF SPIRITUAL WELL-BEING ON SURGICAL FEAR IN PATIENTS SCHEDULED TO HAVE ABDOMINAL SURGERY. International Journal of Health Services Research and Policy 2021;6:229–38. https://doi.org/10.33457/ijhsrp.930665.

[14] Theunissen M, Jonker S, Schepers J, Nicolson NA, Nuijts R, Gramke H-F, et al. Validity and time course of surgical fear as measured with the Surgical Fear Questionnaire in patients undergoing cataract surgery. PLOS ONE 2018;13:e0201511. https://doi.org/10.1371/journal.pone.0201511.

[15] Cancer Registry of Norway. Årsrapport for brystkreft 2021 2022. https://www.kreftregisteret.no/Generelt/Rapporter/Arsrapport-fra-kvalitetsregistrene/Arsrapport-for-brystkreft/arsrapport-for-brystkreft-2021/ (accessed July 19, 2022).

Appendix

Appendix A - Theoretical reasons for in-/excluding certain questionnaires/variables

Explanation: The numbers in the brackets refer to the reference we based the argument for inclusion of the mentioned variable on.

	Based on prior evidence in samples of breast cancer patients	Based on prior evidence on fear of surgery	Based on prior evidence on pre-operative anxiety	Based on theoretical reasoning
**Demographics**		• Educational level: (4 – SFQ-l)<br>• Children: (5)<br>• Marital state: (4)<br>• Occupational status: (4)	• Age: (2)<br>• Gender: (2)<br>• Education: (2) – contradictory	
**Current diagnosis and treatment**	• Neo-adjuvant therapy: (1)	• Mastectomy/Type of surgery: (3)		• DCIS vs. Cancer<br>• Metastases: yes/no<br>• Uni-.vs Bilateral surgery<br>• Type of surgery – each yes/no<br>• SN: yes/no<br>• ALND: yes/no<br>• Hospital stay planned: yes/no
**Medical History**	• Previous cancer: (5)<br>• Psychological comorbidity: (5)	• Prior surgery: (4)<br>• Antidepressant use: (4, SFQ-l)	• Prior surgery: (2)<br>• History of cancer: (2)	
**Clinical outcomes**	• HADS – depression: (1)<br>• HADS – anxiety: (1)<br>• Social Support: (4,5)	• Preoperative pain: (4)<br>• Expected pain: (4)	• Social support: (2)<br>• Injustice experience: (2) as a proxy for feeling of helplessness	• Chronic pain: yes/no<br>• Expected postoperative pain<br>• LOT-R

References based on which the variables were included:

(1) Kyranou M, Puntillo K, Dunn LB, Aouizerat BE, Paul SM, Cooper BA, Neuhaus J, West MC, Dodd M, Miaskowski C. Predictors of initial levels and trajectories of anxiety in women prior to and for six months following breast cancer surgery. Cancer nursing. 2014 Nov;37(6):406.

(2) Stamenkovic DM, Rancic NK, Latas MB, Neskovic V, Rondovic GM, Wu JD, Cattano D. Preoperative anxiety and implications on postoperative recovery: what can we do to change our history. Minerva anestesiologica. 2018 Apr 5;84(11):1307-17

(3) Wittmann V, Márta Csabai MA. *The Healing Effect of Psychosocial Interventions in Breast Cancer-From Surgical Fear to Enhanced Long-term Quality of Life* (Doctoral dissertation, szte).

(4) Theunissen M, Jonker S, Schepers J, Nicolson NA, Nuijts R, Gramke HF, Marcus MA, Peters ML. Validity and time course of surgical fear as measured with the Surgical Fear Questionnaire in patients undergoing cataract surgery. PloS one. 2018 Aug 9;13(8):e0201511.

(5) Singer S, Blettner M, Kreienberg R, Janni W, Wöckel A, Kühn T, Felberbaum R, Flock F, Schwentner L, BRENDA II study group. Breast cancer patients' fear of treatment: results from the Multicenter Longitudinal Study BRENDA II. Breast Care. 2015;10(2):95-100.

(6) Drageset S, Lindstrøm TC, Giske T, Underlid K. Being in suspense: women's experiences awaiting breast cancer surgery. Journal of advanced nursing. 2011 Sep;67(9):1941-51.
