# Supplementary material for: A cross-sectional study of fear of surgery in female breast cancer patients: Prevalence, severity, and sources, as well as relevant differences among patients experiencing high, moderate, and low fear of surgery
Source: PLoS One. 2023 Jun 23;18(6):e0287641. doi: 10.1371/journal.pone.0287641 (PMC10289430; doi:10.1371/journal.pone.0287641)
Supplement: Attachment — Submitted filename: S.Engel_Response to reviewers.pdf [file pone.0287641.s008.pdf]

UiO **Department of Psychology**
University of Oslo

Alejandro Dominguez-Rodriguez, PhD
Academic Editor
PLOS ONE

Date: 6 June 2023

Dear Editor-in-Chief,

Thank you so much for considering and reviewing our research report, '***A cross-sectional study of fear of surgery in female breast cancer patients***: *Prevalence, severity, and sources, as well as relevant differences among patients experiencing high, moderate, and low fear of surgery*', which is as far as we know the first study to systematically investigate fear of surgery among female breast cancer patients.

We are very grateful for the positive feedback we received after the first round of revision. We carefully considered the points raised by reviewer two and adapted our manuscript accordingly (using tracked changes). We genuinely believe that the suggestions improved the quality and clarity of our article.

Already in advance, thank you for your time and for considering our research report. We hope that our revised manuscript will meet the high standards of *PLOS ONE* and are very much looking forward to your response.

Sincerely yours,

Sophia Engel (corresponding author)

On behalf of all the authors

Postal address: Postboks 1094 Blindern, 0317 Oslo
E-mail: ekspedisjonen@psykologi.uio.no
www.sv.uio.no/psi/english/

Line numbers & abbreviations

Line numbers: All line numbers refer to lines in the manuscript including 'tracked changes'.
Abbreviations: SFQ; Surgical Fear Questionnaire

Check of reference list

We reviewed our reference list and find it to be complete. We do not cite any retracted articles.

Reviewer #2 – comments & answers

Comment from reviewer	Answer from author
'I consider that all comments were addressed. However, one last suggestion would be to explicitly state the objective of the study. I suggest add it before the hypotheses.'	Dear reviewer,<br>Thank you so much for your feedback! We agree that the objective of our study could have been stated more clearly. We adapted the introduction according to your suggestion (line 90-93). We hope that the changes we have made address your feedback adequately.<br>Thank you so much!
'Have the authors made all data underlying the findings in their manuscript fully available? No.'	As indicated in the data availability statement in the submission history, it is unfortunately not possible to make the data underlying our manuscript fully available yet. As our study uses privacy sensitive patient data, data need to be anonymized before they can be accessed. Since the cross-sectional data analyzed in the present study stem from a larger longitudinal trial, the PREVENT trial, data will only be anonymized and made accessible after the trial is completed. This is also described in the PREVENT trial's protocol which was published in PLOS One as well [1]. However, to increase the transparency, we published the 'R' script underlying the analyses of the present study. Moreover, as data collection in the frame of the PREVENT trial has been completed in March 2023 the anonymized data will be made available in the close future and can be accessed as described in the data availability statement.<br><br>We hope the above gives a satisfactory overview of the reasons that keep us from publishing the data underlying our manuscript at this moment.

References

1. Reme SE, Munk A, Holter MTS, Falk RS, Jacobsen HB. Pre- and post-operative psychological interventions to prevent pain and fatigue after breast cancer surgery (PREVENT): Protocol for a randomized controlled trial. PLOS ONE. 2022;17: e0268606. doi:10.1371/journal.pone.0268606
